# Tumor immune contexture is a determinant of anti-CD19 CAR T cell efficacy in large B cell lymphoma

**DOI:** 10.1038/s41591-022-01916-x

**Published:** 2022-08-29

**Authors:** Nathalie Scholler, Regis Perbost, Frederick L. Locke, Michael D. Jain, Sarah Turcan, Corinne Danan, Edmund C. Chang, Sattva S. Neelapu, David B. Miklos, Caron A. Jacobson, Lazaros J. Lekakis, Yi Lin, Armin Ghobadi, Jenny J. Kim, Justin Chou, Vicki Plaks, Zixing Wang, Allen Xue, Mike Mattie, John M. Rossi, Adrian Bot, Jérôme Galon

**Affiliations:** 1 Kite, a Gilead company, Santa Monica, CA USA; 2 Gilead Sciences, Foster City, CA USA; 3 Veracyte SAS, Marseille, France; 4 Moffitt Cancer Center, Tampa, FL USA; 5 The University of Texas MD Anderson Cancer Center, Houston, TX USA; 6 Stanford University School of Medicine, Stanford, CA USA; 7 Dana-Farber Cancer Institute, Boston, MA USA; 8 University of Miami Health System, Sylvester Comprehensive Cancer Center, Miami, FL USA; 9 Mayo Clinic, Rochester, MN USA; 10 Washington University School of Medicine, St. Louis, MO USA; 11 Capstan Therapeutics, San Diego, CA USA; 12 INSERM, Sorbonne Université, Université Paris Cité, Centre de Recherche des Cordeliers, Equipe Labellisée Ligue Contre le Cancer, Laboratory of Integrative Cancer Immunology, Paris, France

**Keywords:** Tumour immunology, Cancer microenvironment, Translational research, Immunotherapy

## Abstract

Axicabtagene ciloleucel (axi-cel) is an anti-CD19 chimeric antigen receptor (CAR) T cell therapy approved for relapsed/refractory large B cell lymphoma (LBCL) and has treatment with similar efficacy across conventional LBCL subtypes. Toward patient stratification, we assessed whether tumor immune contexture influenced clinical outcomes after axi-cel. We evaluated the tumor microenvironment (TME) of 135 pre-treatment and post-treatment tumor biopsies taken from 51 patients in the ZUMA-1 phase 2 trial. We uncovered dynamic patterns that occurred within 2 weeks after axi-cel. The biological associations among Immunoscore (quantification of tumor-infiltrating T cell density), Immunosign 21 (expression of pre-defined immune gene panel) and cell subsets were validated in three independent LBCL datasets. In the ZUMA-1 trial samples, clinical response and overall survival were associated with pre-treatment immune contexture as characterized by Immunoscore and Immunosign 21. Circulating CAR T cell levels were associated with post-treatment TME T cell exhaustion. TME enriched for chemokines (CCL5 and CCL22), γ-chain receptor cytokines (IL-15, IL-7 and IL-21) and interferon-regulated molecules were associated with T cell infiltration and markers of activity. Finally, high density of regulatory T cells in pre-treatment TME associated with reduced axi-cel–related neurologic toxicity. These findings advance the understanding of LBCL TME characteristics associated with clinical responses to anti-CD19 CAR T cell therapy and could foster biomarker development and treatment optimization for patients with LBCL.

## Abstract (web summary)

Analysis of tumor biopsies from the pivotal phase 1/2 ZUMA-1 trial identifies pre-treatment T cell–related characteristics that are associated with clinical response and neurologic toxicity after anti-CD19 CAR T cell therapy in patients with large B cell lymphoma.

## Main

Immunotherapies have revolutionized cancer treatment^1–4^. Axicabtagene ciloleucel (axi-cel) is a first-in-class anti-CD19 CAR T cell therapy approved for the treatment of relapsed/refractory (r/r) LBCL. In the pivotal ZUMA-1 study (NCT02348216), the objective response (OR) rate was 83% (58% complete response (CR) rate); 39% of patients had ongoing responses (median of 27.1 months of follow-up); and grade ≥3 neurologic events (NEs) were reported in 32% of patients^5^. Unlike other therapies, such as rituximab^6,7^, axi-cel has similar efficacy across LBCL subtypes defined through conventional histological, cytogenetic and molecular prognostic markers^7,8^. However, ~60% of patients showed primary treatment resistance (~15%) or relapse (~45%) within the first year^5^, warranting efforts to understand mechanisms and markers underlying response.

Although the prognostic and predictive roles of the TME^9–15^ have been described for solid tumors, including for checkpoint inhibitors^16–23^, the importance of TME for CAR T cell therapy has not been established. Taking into account the immune mechanistic nature of the CAR T cell intervention and its proven activity in patients with diffuse LBCL (DLBCL) with poor or favorable conventional tumor-related prognostic markers, such as bcl-2, bcl-6 and c-Myc status, we first hypothesized that dynamic patterns developing rapidly in the post-treatment TME distinguish responders and non-responders and, second, that certain characteristics of the tumor immune contexture, pre-treatment, may associate with CAR T cell treatment outcome. Specifically, features of tumor immune contexture, such as presence of activated or exhausted T cells, infiltration with immune regulatory myeloid cells or other categories of immune cells, along with gene expression programs that may influence the recruitment, expansion and activity of T cells, may, in fact, provide a global integrative perspective on whether the given status of the tumoral process and immune system in a given patient are favorable or detrimental with respect to benefitting from this treatment modality.

**Extended Data Fig. 1:**
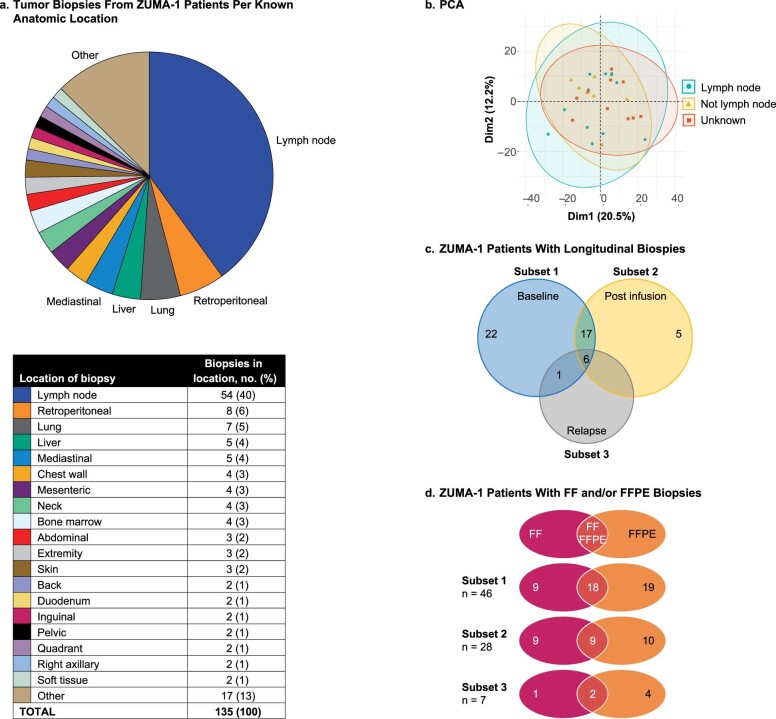
Characterization of ZUMA-1 tumour biopsy specimens analysed in this study. a, Number of tumour biopsies per known anatomic location from Zuma 1 patients analysed in the manuscript; b, PCA of Zuma 1 baseline biopsies (subset 1) with the two first principal components of all gene expression. Data are colored by biopsy origin; lymph node, blue, n = 12; not lymph node origin (as indicated in a), yellow, n = 7; unknown origin, red, n = 10; c. Number of Zuma 1 patients analyzed in the manuscript, classification per subset, and overview of the longitudinal biopsies; d. Number of Zuma 1 patients analyzed in the manuscript with FF and/or FFPE biopsies classified per subset. FF, fresh frozen; FFPE, formalin-fixed paraffin embedded; PCA, principal component analysis.

In this study, we systematically investigated a broad range of immune programs in pre-treatment and post-treatment biopsy specimens from the ZUMA-1 (refs. ^5,24^) pivotal study (Supplementary Table 1 and Extended Data Fig. 1) and uncovered key TME immune features that associate with clinical outcomes. When possible, we validated our findings in three independent datasets—namely, treatment-naive biopsy specimens and pre-treatment specimens from patients enrolled in an ongoing, second-line CAR T cell interventional study^25^ (ZUMA-7; NCT03391466) and from patients treated with commercial axi-cel at Moffitt Cancer Center^26^ (Supplementary Tables 2 and 3). These findings advance understanding of the effect of CAR T cell therapy on the TME and the association with clinical response; they also foster biomarker development and treatment optimization.

## Results

### TME rapid evolution was a hallmark of clinical response

**Fig. 1:**
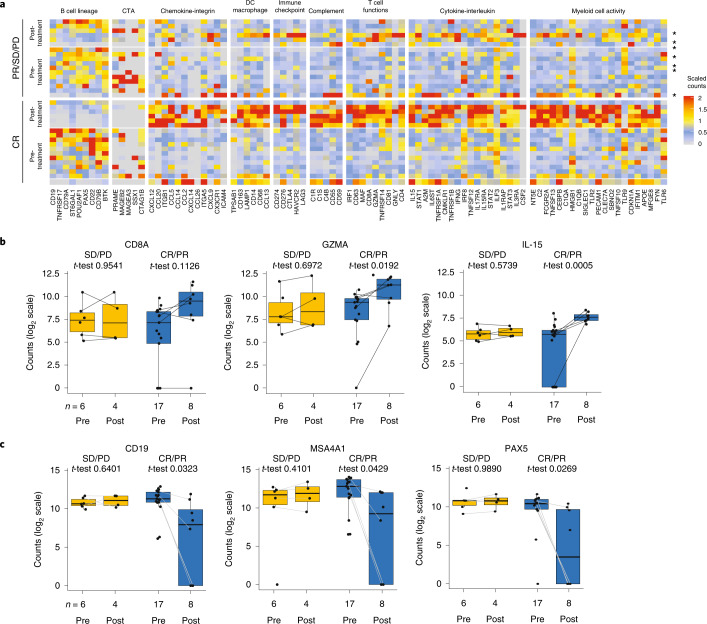
Evolution of TME after axi-cel infusion associated with clinical outcomes in ZUMA-1. **a**, Heat map of gene expression measured by PanCancer Immune Profiling panel (NanoString) in FF tumor biopsy specimens from patients in ZUMA-1 at baseline (before conditioning and axi-cel infusion, subset 1, *n* = 23) and 2–4 weeks after axi-cel infusion (subset 2, *n* = 13). Among the genes with significant differential expression between pre-treatment and post-treatment (two-sided *t*-test without adjustment, *P* < 0.05), shown genes were selected according to their belonging to a specific TME-related signature. Patients with CR (*n* = 18 (12 pre-treatment; six within 2 weeks post-treatment)), PR (*n* = 7, as noted by asterisks (five pre-treatment, two within 2 weeks post-treatment)) and SD/PD (*n* = 11 (six pre-treatment, four within 2 weeks post-treatment and one with SD within 4 weeks post-treatment) are shown. The color range is set to log-transform scaled values. Scaled values are calculated by dividing by the standard deviation. **b**,**c**, Expression of T cell–related genes (**b**) and B cell lineage genes (**c**) measured by PanCancer Immune Profiling panel in paired FF biopsy specimens. Values and two-sided *t*-test without adjustment in embedded tables. DC, dendritic cell; FOXP3, forkhead box P3; GZMA, granzyme A; IL, interleukin; PAX5, paired box protein 5; PD, progressive disease; PD-1, programmed cell death protein 1; PR, partial response; SD, stable disease.

**Extended Data Fig. 2:**
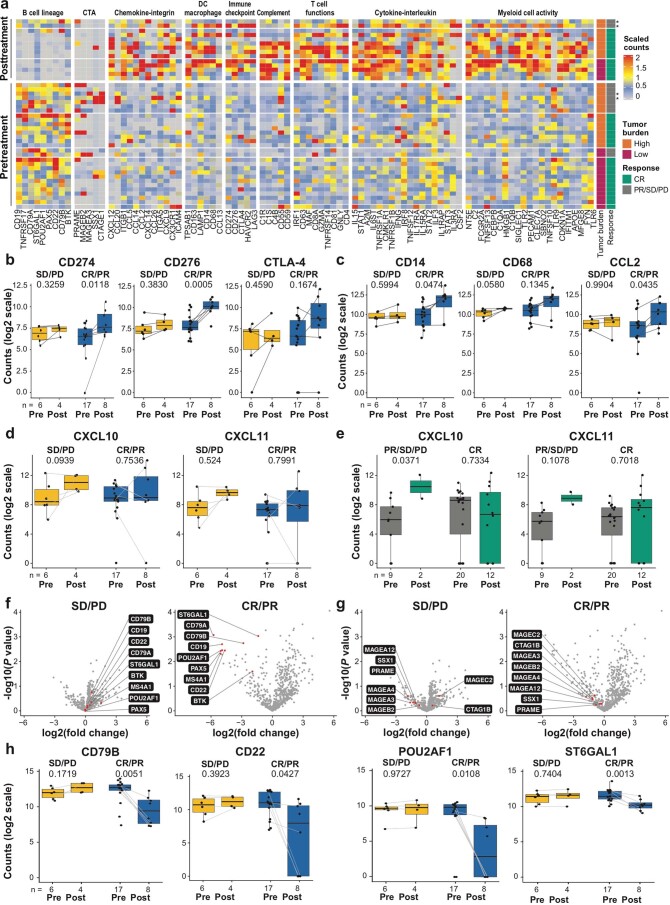
Evolution of the tumour infiltrate in ZUMA-1 patients after axi-cel infusion associated with clinical outcomes independently of tumour burden. a, Heatmap of gene expression measured by PanCancer Immune Profiling panel (NanoString) in FFPE tumour biopsy specimens pretreatment (subset 1, n = 29) or posttreatment (subset 2, n = 14) from Zuma 1 patients with high or low tumour burden at pretreatment. Among the genes with significant differential expression between pre and post treatment (Two-sided t.test without adjustment, p.value <0.05), shown genes were selected according to their belonging to a specific TME related signature. Patients with CR and low tumour burden, n = 17 (Subset 1, n = 12; Subset 2, n = 5); with CR and high tumour burden, n = 15 (Subset 1, n = 8; Subset 2, n = 7); without CR and low tumour burden, n = 2 (Subset 1); without CR and high tumour burden, n = 9 (Subset 1, n = 7; Subset 2, n = 2). PR (n=4, as noted by asterisks [2 pretreatment; 2 within 2 weeks posttreatment]. The color range is set to log-transform scaled values. Scaled values are calculated by dividing by the standard deviation. b-e, Gene expression measured by PanCancer Immune Profiling panel in paired specimens; (two sided t.test without adjustment); b,c, T-cell functional genes (b) and myeloid- and cytokine-related genes (c) in FF biopsies; d,e, CXCL10 and CXCL11 genes in FF (d) vs. FFPE (e) biopsies. *P < 0.1 and ***P < 0.01. f,g, Volcano plots of gene expression from pre- vs. posttreatment biopsies of patients with SD/PD or CR/PR (as indicated) for B-cell lineage (f) and CTA genes (g). The plots were constructed using log2(fold change) and log10 (P values) for all genes. Red dots represent the top differentially expressed genes with P<0.01. h, Comparative gene expression of B-cell lineage selected markers in pre- vs posttreatment biopsies. *P < 0.1 and **P < 0.05.(Two-sided t.test without adjustment). axi-cel, axicabtagene ciloleucel; CCL, chemokine ligand; CR, complete response; CTA, cancer testis antigen; CTLA-4, cytotoxic T lymphocyte–associated protein 4; CXCL, chemokine C-X-C motif ligand; DC, dendritic cell; MAGE-B2, melanoma-associated antigen B2; PD, progressive disease; PD-L1, programmed death-ligand 1; PF: Fresh frozen; FFPE, formalin-fixed paraffin-embedded; POU2AF1, POU domain class 2-associating factor 1; PR, partial response; SD, stable disease.

Best responses to CAR T cell therapy include rapid CAR expansion within 7–14 days after infusion and anti-tumor activity typically evaluated by positron emission tomography–computed tomography (PET–CT) 30 days after infusion. However, the lack of robust predictive biomarkers for patient stratification and monitoring remains a major challenge in the field of cellular immunotherapy. To investigate mechanisms and markers underlying response to axi-cel, we analyzed and compared pre-treatment TME patterns that may distinguish responders from non-responders by transcriptomics (Supplementary Tables 4 and 5) and assessed whether axi-cel triggered changes of the tumor immune contexture detectable before the first evaluation of clinical response, which is typically evaluated 1 month after axi-cel infusion by PET–CT imaging. Figure 1 and Extended Data Fig. 2 highlight the significant transcriptomics changes in tumor biopsies from pre-treatment to 2 weeks after axi-cel in function of the therapeutic response. Supplementary Tables 6–8 provide the differential expression of all the genes assessed before and after axi-cel infusion per therapeutic response type. In responders, the post-treatment evolution of TME gene expression patterns strikingly differed from those of non-responders; results were consistent across fresh-frozen (FF; Fig. 1a,b) and formalin-fixed paraffin-embedded (FFPE) sample sets (Extended Data Fig. 2) and were independent of pre-treatment tumor burden (TB; Extended Data Fig. 2a).

Differences spanned all major categories of TME genes, including innate and adaptive immunity and tumor-related and stroma-related genes with well-described immune activity or yet-to-be-determined functionality (Supplementary Tables 6–8). Responders showed early and rapid elevation of cytotoxic T cell–related genes (CD8α and granzyme A) and key T cell growth factor (IL-15) (Fig. 1a,b) as well as interferon (IFN)-γ–regulated immune checkpoint (IC) encoding genes (CD274, CD276 and CTLA-4), myeloid-related genes and chemokines (CD14, CD68 and CCL2) (Extended Data Fig. 2b,c). The evolution of TME gene expression patterns was vastly different in non-responders, where no increase in immune-related genes was observed, except for a positive trend in two pro-inflammatory chemokines (CXCL10 and CXCL11) that was consistent across FF and FFPE sample sets (Extended Data Fig. 2d,e) and likely modulated by chemo-conditioning^27^.

Gene expression across all B cell lineage markers decreased markedly in the TME from responders, including CD19, CD20 (ref. ^28^), B cell transcriptional master switch PAX5 (ref. ^29^) (Fig. 1c), ST6GAL1 (ref. ^28^), CD22 and transcriptional coactivator POU2AF1 (ref. ^30^) (Extended Data Fig. 2f,h). These changes likely reflected axi-cel–mediated tumor cell clearance given that patients in ZUMA-1 were largely B cell aplastic at enrollment due to prior rituximab treatment^24^. In addition, the rapid decrease in gene expression of cancer testis antigens (CTAs^31^) was more marked in responders (Extended Data Fig. 2g), further supporting a rapid evolution toward a reduced tumor-related signature consistent with the negative correlation in DLBCL between tumor-infiltrating immune cell signature and B cell signature (for example, CD19, MS4A1, CD79A and CD79B)^32^. Finally, these changes were similar across strata defined by classical prognostic markers^24^, and, whereas many immune contexture genes were strongly upregulated, other immune genes specific for other immune cell types, including TLR9, HMGB1, ILF3, CSF2, IL3RA and ICAM4, were not increased post-treatment. Altogether, rapid and broad changes across adaptive and innate immune programs and B cell lineage markers in post-treatment TME distinguished responders from non-responders.

### T cell density in TME associated with CR

**Fig. 2:**
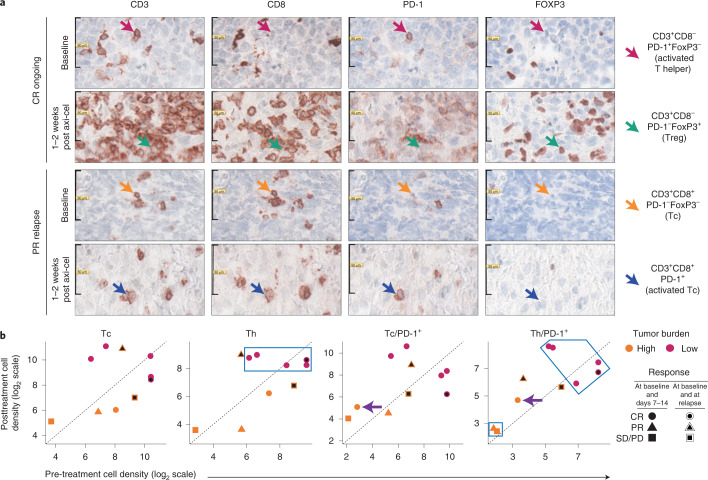
Evolution of T cell subset densities in the TME after axi-cel infusion. **a**, Representative T cell subset densities measured by Immunoscore TCE/TCE^+^ in patients with ongoing CR versus PR with relapse before and after axi-cel infusion (FFPE biopsy specimens, one staining per sample per marker). **b**, Evolution of T cell subset densities in the TME as a function of clinical response and TB. Analysis by Immunoscore TCE/TCE^+^ of FFPE biopsy specimens from ten patients in ZUMA-1 with high (>3,000 mm^2^; *n* = 5) or low (<3,000 mm^2^; *n* = 5) TB. Seven of the paired samples belonged to subsets 1 and 2, and three belonged to subsets 1 and 3. Six patients had CR; two patients had PR; and two patients had SD/PD. In the Th panel, the blue square indicates five patients with CR with low TB and high Th cell density within the TME pre-infusion and post-infusion (50% of tested patients, two-sided exact Fisher test, *P* = 0.10). One patient relapsed >6 months after initial CR (circle with black center). The purple arrows in the Tc/PD-1^+^ and Th/PD-1^+^ panels indicate a patient with CR despite high TB and with increased PD-1^+^ tumor-infiltrating T cells in post-treatment TME (10% of tested patients). The black square in the Th/PD-1^+^ panel indicates non-CR patients with high TB and low CD4^+^ T cell density pre-treatment and post-treatment (20% of tested patients, Fisher test, *P* = 0.09). Data are also shown for two patients (one with PR and one with SD/PD) with high TB and intermediary/high tumor-infiltrating T cell density post-infusion who both relapsed (20% of tested patients, Fisher test, *P* = 0.10). FOXP3, forkhead box P3; PD, progressive disease; PD-1, programmed cell death protein 1; PR, partial response; SD, stable disease.

**Extended Data Fig. 3:**
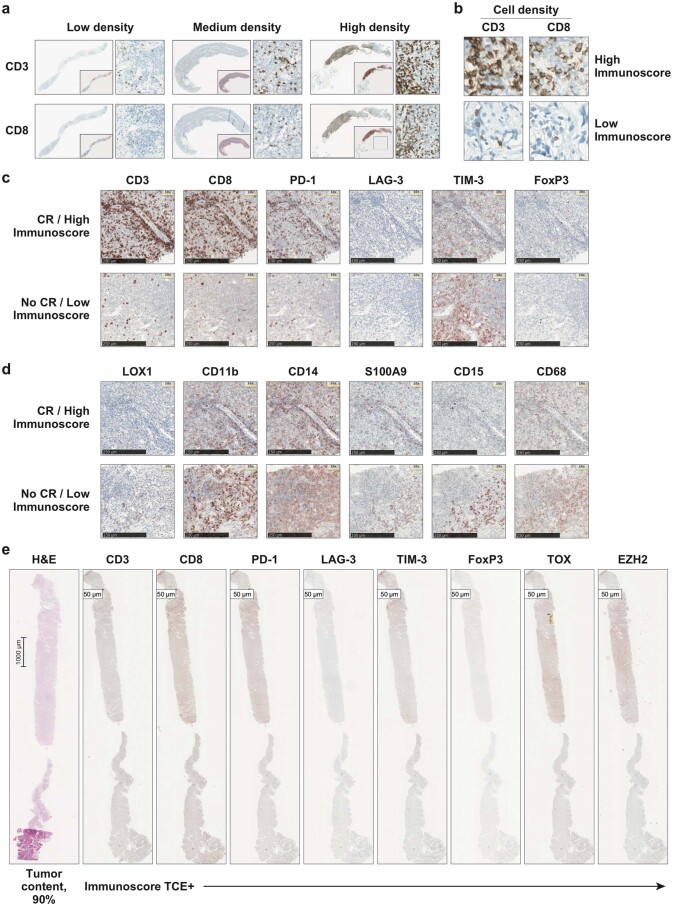
Overview of Immunoscore index, Immunoscore TCE/TCE+ panels, and Immunoscore SC panel. a,b, Following the pathologist selection of a digital image area, IHC staining of CD3 and CD8 was quantified by the application of a prespecified bioinformatics algorithm that generated analysis cutoffs and a numerical index named Immunoscore. Because the majority of lymph node biopsy specimens lacked an identifiable invasive margin, as expected for lymphoma, cell densities were calculated from the core tumour only. a, Representative images (250- and 1000-µm scales) of CD3 and CD8 T-cell densities (low, medium, or high) in tumour biopsy specimens. b, Representative images (50- µm scale) of CD3 and CD8 T-cell densities in patients with a high (top panels) or low (bottom panels) Immunoscore index. c,d, Representative images (250-µm scale) of successive stainings with Immunoscore TCE (c) and Immunoscore SC (d) panels of tumour biopsy specimens from patients in ZUMA-1 with CR (top panels) or without CR (bottom panels). e, Representative images (50-µm scale) of stainings with Immunoscore TCE+ panel of tumour biopsy specimens. One staining per sample per marker. EZH2, enhancer of zeste homolog 2; FOXP3, forkhead box P3; H&E, haematoxylin and eosin; IHC, immunohistochemistry; LAG-3, lymphocyte-activation gene 3; LOX-1, lectin-type oxidised LDL receptor 1; PD-1, programmed cell death protein 1; SC, Suppressive Cell; TCE, T-cell Exhaustion; TIM-3, T-cell immunoglobulin and mucin domain 3; TOX, thymocyte selection–associated high mobility group box.

**Extended Data Fig. 4:**
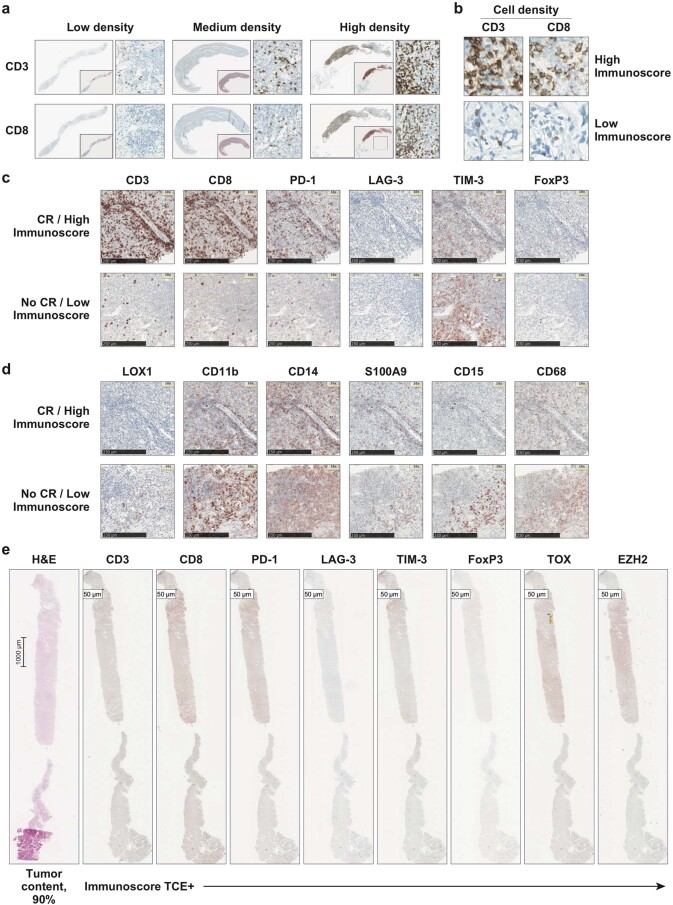
Overview of Immunoscore TCE+ panel. a, Representative images (50-µm scale; 40× magnification) of staining with Immunoscore TCE+ panel of ZUMA-1 tumour biopsy specimens from patients with ongoing CR versus relapse after PR, at pre- (top panels) and posttreatment (bottom panels). b, Representative images (50-µm scale; 40× magnification) of cell phenotyping with Immunoscore TCE+ panel in ZUMA-1 tumour biopsy specimens. Arrows point to helper T-cells (CD3+CD8–PD-1+LAG-3+TIM-3–FoxP3–TOX–EZH2+), cytotoxic T-cells (CD3+CD8+PD-1+LAG-3–TIM-3+FoxP3–TOX–EZH2+), regulatory T-cells (CD3+CD8–PD-1+LAG-3+/–TIM-3–FoxP3+TOX–EZH2+), exhausted helper T-cells (CD3+CD8–PD-1+LAG-3+TIM-3–FoxP3–TOX+EZH2+), and tumour cells (EZH2+CD3–). One staining per sample per marker. axi-cel, axicabtagene ciloleucel; CR, complete response; EZH2, enhancer of zeste homolog 2; FOXP3, forkhead box P3; LAG-3, lymphocyte-activation gene 3; PD-1, programmed cell death protein 1; PR, partial response; TCE, T-cell Exhaustion; TIM-3, T-cell immunoglobulin and mucin domain 3; TOX, thymocyte selection-associated high mobility group box.

**Extended Data Fig. 5:**
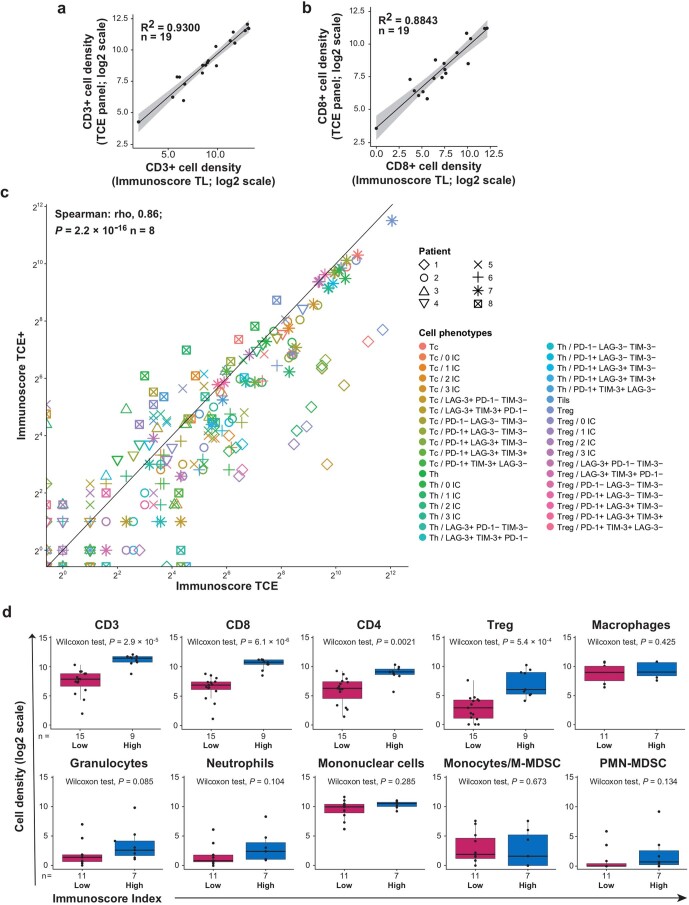
Correlative analyses with Immunoscore panels. a,b, Correlations of CD3+ (a) and CD8+ (b) T-cell densities measured with Immunoscore TL (X-axis) versus Immunoscore TCE (Y-axis) panels in ZUMA-1 patient biopsies at pretreatment (subset 1, n = 19). The grey ribbons represent the 95% Confidence Interval of the regression line. c, Correlation between cell phenotypes measured with Immunoscore TCE (X-axis) versus Immunoscore TCE+ (Y-axis) panels in two adjacent tissue slides (1 slide per panel) from ZUMA-1 patient biopsies (subset 1, n = 8). Statistical significance of the spearman coefficient level (two-sided P value) as shown. d, Association between cell densities of tumour-infiltrating immune subsets (T-cells or myeloid cells, as indicated) and Immunoscore index (low versus high) in ZUMA-1 patient biopsies (subset 1, n = 24 and n = 18, respectively). Two-sided Wilcoxon test, p values as shown. ABC, activated B-cell DLBCL subtype; DLBCL, diffuse large B-cell lymphoma; GCB, germinal centre B-cell DLBCL subtype; LAG-3, lymphocyte-activation gene 3; IC, immune checkpoint; M-MDSC, monocytic myeloid-derived suppressor cell; PD-1, programmed cell death protein 1; PMN-MDSC, polymorphonuclear myeloid-derived suppressor cell; Tc, cytotoxic T-cell; TCE, T-cell Exhaustion; Th, helper T-cell; TIM-3, T-cell immunoglobulin and mucin domain 3; Treg, regulatory T-cell.

To validate these findings at the cellular and protein level, we performed multiplex spatial tissue analyses that combine multiplexed immunohistochemistry (IHC) (Fig. 2a and Extended Data Figs. 3–5), advanced image analysis and computerized algorithm^33^. This methodology was initially developed to create the Immunoscore index^20^ to quantify tumor T cell infiltrate and better predict the prognosis of patients with colorectal cancer than AJCC/UICC TNM staging. Tumor immune contexture^22^ characterizes the spatial organization and density of tumor-infiltrating immune cells and predicts prognosis and response to IC inhibitors in various solid tumors. To adapt the approach to r/r LBCL and to evaluate additional immune cell subsets and exhaustion markers, we developed three panels in addition to Immunoscore T lymphocytes (TLs): Immunoscore T cell exhaustion (TCE) and TCE^+^ (with TOX marker) panels and Immunoscore suppressive cells (SCs) panel (Extended Data Figs. 3 and 4 and Supplementary Table 9). We demonstrated excellent consistency in quantification of CD3^+^ and CD8^+^ T cell densities between Immunoscore TL and Immunoscore TCE (Extended Data Fig. 5a,b) and between Immunoscore TCE and Immunoscore TCE^+^ (Extended Data Fig. 5c). Lastly, the Immunoscore index significantly correlated with tumor infiltration of T cell subsets, including CD3, CD4, CD8 and regulatory T cells (Tregs), but not with myeloid cell subsets in pre-treatment ZUMA-1 biopsies (Extended Data Fig. 5d).

Using these panels, we first investigated whether T cell density in pre-treatment and/or post-treatment TME associated with clinical outcomes and TB. Immunoscore TCE was measured in ten paired samples from the ZUMA-1 subset 1 (pre-treatment) and subset 2 (early post-treatment) biopsies. Figure 2b shows that all patients with low TB and higher helper T (Th) cell density achieved CR (pink circles in blue squares), and their TME presented a higher T cell density both pre-therapy and post-therapy to compare with the TME from patients with high TB (orange symbols). Conversely, four of five patients with pre-treatment high TB and low T cell density did not achieve CR. However, one patient with high TB achieved CR. This patient (orange circle) presented a pre-treatment intermediary T cell density with post-treatment evolution of TME to higher density of T cells with activated phenotype (PD1^+^LAG-3^−^TIM3^−^) (purple arrow). The results of this small dataset suggest that post-treatment TME T cell density reflected pre-treatment density and supported CR association with low TB and high cell density before the first clinical response evaluation.

### Lower CAR T levels associated with TME exhausted T cells

Early studies showed that axi-cel efficacy associates with rapid CAR T cell expansion in blood^5,24^ and that the ratio of early CAR T cell expansion to pre-treatment TB associates with durable response^34^. We hypothesized that tumor immune contexture pre-treatment and early post-treatment could better predict LBCL response to axi-cel than classical prognostic markers (described in ref. ^24^; Supplementary Fig. 3a), including the activated B cell–like (ABC) cell-of-origin (COO) subtype that predicts a lower overall survival after standard chemotherapy^35,36^ than germinal center B cell–like (GCB) or unclassified subtypes of DLBCL.

**Fig. 3:**
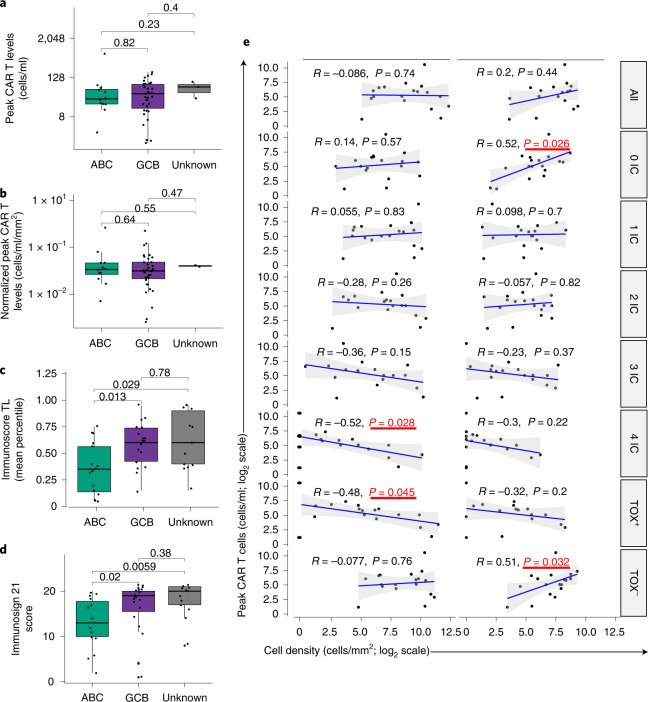
Correlations between circulating CAR T cell levels and tumor immune contexture. **a**–**d**, COO subtypes were determined by independent transcriptomics analyses in ZUMA-1 samples (*n* = 51) (**a**,**b**) and commercially available DLBCL biopsies (**c**,**d**) at diagnosis (*n* = 50, 16/20/14 for ABC/GCB/UN, respectively). COO subtypes were correlated to peak CAR T cell levels (two-sided *t*-test without adjustment *P* values as shown) absolute (**a**) or normalized (**b**) to TB and to Immunoscore TL (**c**) and Immunosign 21 (**d**). **e**, Peak CAR T cell levels were also correlated to cell densities of tumor-infiltrating Tc or Th measured with Immunoscore TCE^+^ panel in ZUMA-1 patient biopsies, 7–14 days after axi-cel infusion (subset 2, *n* = 18). Tc and Th phenotypes were classified by checkpoint expression (PD-1^+/–^, LAG-3^+/–^, TIM-3^+/–^ and TOX^+/–^). The gray ribbons represent the 95% confidence interval of the regression line. Statistical significance of the Spearman coefficient level (two-sided *P* value) was calculated, and significant *P* values (<0.05) are shown in red squares. From top to bottom: all phenotypes (any checkpoint), 0 IC (no checkpoint), 1 IC (any one checkpoint: PD-1 or LAG-3 or TIM-3 or TOX), 2 IC (any two checkpoints: PD-1^+^LAG-3^+^ or PD-1^+^TIM-3^+^ or PD-1^+^TOX^+^ or LAG-3^+^TIM-3^+^ or LAG-3^+^/TOX^+^ or TIM-3^+^TOX^+^), 3 IC (any three checkpoints: PD-1^+^LAG-3^+^TIM-3^+^ or PD-1^+^LAG-3^+^TOX^+^ or LAG-3^+^TIM-3^+^TOX^+^), 4 IC (all four checkpoints: PD-1^+^LAG-3^+^TIM-3^+^TOX^+^), TOX^+^ (TOX^+^ in combination with any checkpoint(s): TOX^+^PD-1^+/–^LAG-3^+/–^TIM-3^+/–^) and TOX^–^ (any combination of checkpoint(s) without TOX: TOX^–^PD-1^+/–^LAG-3^+/–^TIM-3^+/–^). LAG-3, lymphocyte activation gene 3; PD-1, programmed cell death protein 1; TIM-3, T cell immunoglobulin and mucin domain 3; TOX, thymocyte selection–associated high mobility group box.

**Extended Data Fig. 6:**
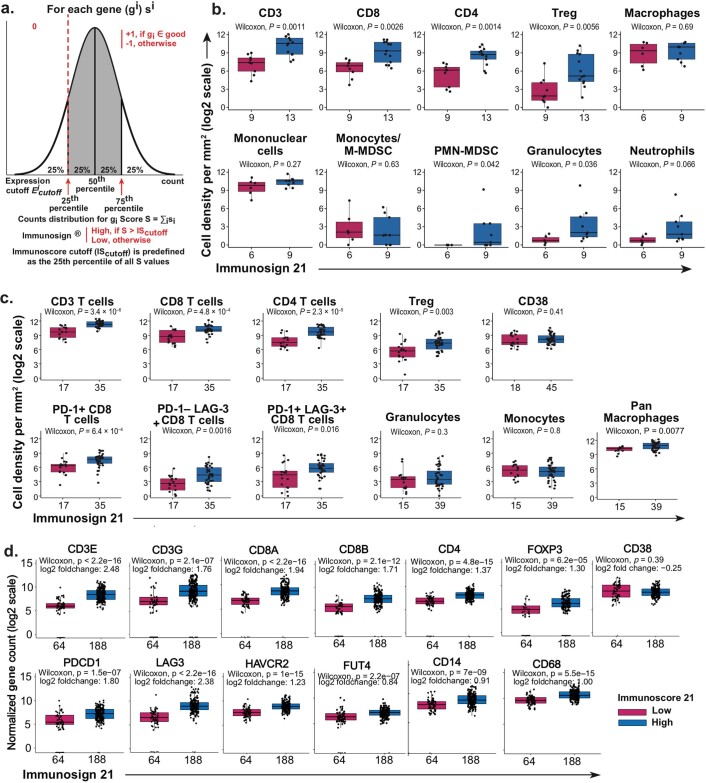
Correlative studies of Immunosign 21 score with cell subsets across 3 independent datasets. a, Definition of the Immunosign 21 score cutoff. The Immunosign 21 scoring function is an algorithm derived from the Immunoscore algorithm, is independent of clinical outcome, and is predefined as the 25th percentile of the observed scores among samples. b-d, Correlation of Immunosign 21 (low versus high) with cell subsets in 3 independent datasets (Two-sided Wilcoxon test). b,c, Immunosign 21 correlations with cell subset densities analysed by Immunoscore TCE/TCE+ panels in b, ZUMA-1 pretreatment tumour biopsy specimens from third line, r/r DLBCL patients (subset 1, n = 22 for T-cell subsets; n = 15 for myeloid subsets) and c, DLBCL biopsy specimens at diagnosis from treatment-naïve patients (n = 67). d, Immunosign 21 correlations with normalised gene expression of T-cells or of myeloid cells analysed by IO360 NanoString panel in pretreatment tumour biopsy specimens from second line DLBCL patients (n = 252). The number of samples per group is indicated on each panel. DLBCL, diffuse large B-cell lymphoma; FOXP3, forkhead box P3; LAG-3, lymphocyte-activation gene 3; M-MDSC, monocytic myeloid-derived suppressor cell; PD-1, programmed cell death protein 1; PMN-MDSC, polymorphonuclear myeloid-derived suppressor cell; SC, Suppressive cell; TCE, T-cell Exhaustion; TIM-3, T-cell immunoglobulin and mucin domain 3; Treg, regulatory T-cell.

**Extended Data Fig. 7:**
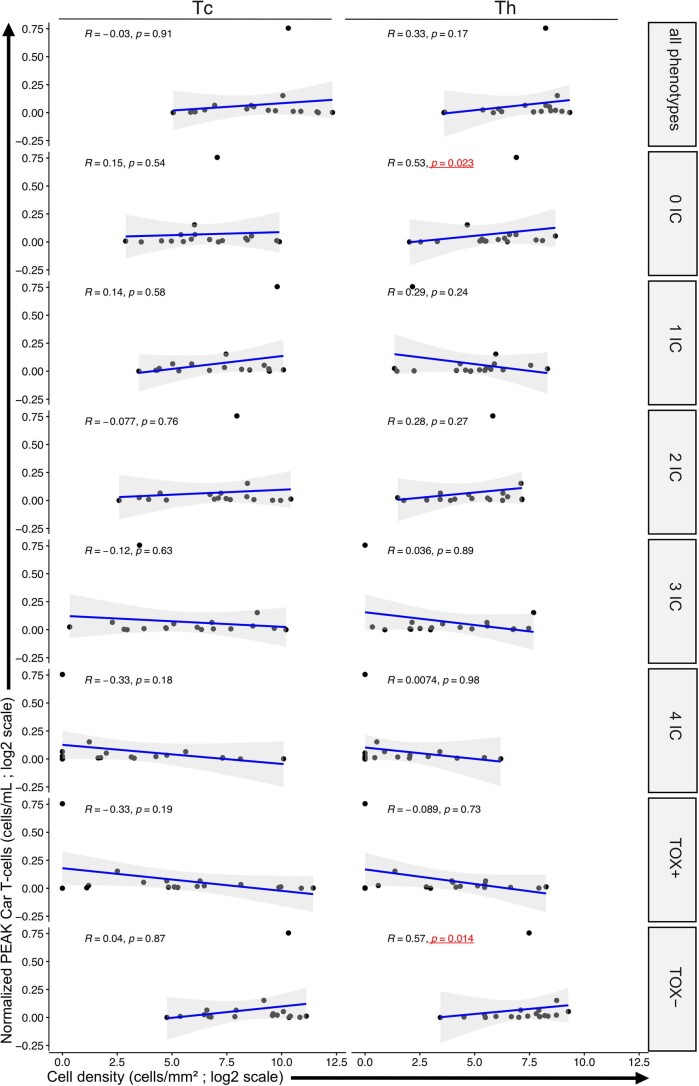
Circulating peak CAR T-cell levels normalised to pretreatment tumour burden correlated to Immunoscore. T-cell densities were measured with Immunoscore TCE+ panel in ZUMA-1 patient biopsies, 7 to 14 days after axi-cel infusion (Subset 2, n = 18). Tc (left panels) and Th (right panels) phenotypes were classified by checkpoint expression (PD-1+/–, LAG-3+/–, TIM-3+/–, TOX+/–). The grey ribbon represents the 95% Confidence Interval of the regression line. Statistical significance of the spearman coefficient level (two-sided P values) were calculated and significant p values (< 0.05) are shown in red squares. From top to bottom: all phenotypes (any checkpoint), 0 IC (no checkpoint), 1 IC (any 1 checkpoint: PD-1 or LAG-3 or TIM-3 or TOX), 2 IC (any 2 checkpoints: PD-1+/LAG-3+ or PD-1+/TIM-3+ or PD-1+/TOX+ or LAG-3+/TIM-3+ or LAG-3+/TOX+ or TIM-3+/TOX+), 3 IC (any 3 checkpoints: PD-1+LAG-3+TIM-3+ or PD-1+LAG-3+TOX+ or LAG-3+TIM-3+TOX+), 4 IC (all 4 checkpoints: PD-1+LAG-3+TIM-3+TOX+), TOX+ (TOX+ in combination with any checkpoint(s): TOX+PD-1+/–LAG-3+/–TIM-3+/–), TOX– (any combination of checkpoint(s) without TOX: TOX–PD-1+/–LAG-3+/–TIM-3+/–), and TOX only (TOX+PD-1–LAG-3–TIM-3–). axi-cel, axicabtagene ciloleucel; CAR, chimeric antigen receptor; LAG-3, lymphocyte-activation gene 3; IC, immune checkpoint; PD-1, programmed cell death protein 1; Tc, cytotoxic T-cell; Th, helper T-cell; TIM-3, T-cell immunoglobulin and mucin domain 3; TME, tumour microenvironment; TOX, thymocyte selection–associated high mobility group box.

We, thus, investigated whether there was an association between circulating CAR T cell levels and pre-treatment and post-treatment tumor immune contexture to compare with COO. Tumor immune contexture was evaluated with Immunoscore TL, TCE^+^ and Immunosign 21 (ref. ^37^). Immunosign 21 profiles the expression of a pre-defined set of genes associated with T cell and innate immune programs, effector T cells, ICs, chemokines and IFN-related molecules (Supplementary Table 5). Immunosign 21 score captured immune contexture-related features in all (three) independent datasets analyzed (*n* = 341 patients; Extended Data Fig. 6) and was highly correlated with Immunoscore both in the ZUMA-1 subset and therapy-naive DLBCL from commercial origin (Supplementary Fig. 1a,b). Immunoscore trended positively with axi-cel response (Supplementary Fig. 1d–j). In agreement with previous findings showing similar axi-cel efficacy across disease subtypes and stages^6–8^, we found no association between peak CAR T cell levels and COO (Fig. 3a,b). However, Immunoscore TL index and Immunosign 21 score were significantly lower in the ABC COO subtype (Fig. 3c,d), suggesting that TME-independent features may contribute to therapeutic response and compensate for TME detrimental characteristics. In fact, circulating peak CAR levels associated positively with post-treatment TME density of Th cells lacking expression of ICs or of TOX, a known marker of T cell exhaustion^28^, before (Fig. 3e, right panel) and after normalization to TB (Extended Data Fig. 7). Consistent with this observation, the density of cytotoxic T (Tc) cells expressing all four ICs or TOX^+^ in combination with any checkpoint associated negatively with circulating CAR T cell levels in post-treatment TME (Fig. 3e, left panel). These results revealed an association between poor CAR T cell expansion in blood and post-treatment TME infiltration with exhausted Tc cells. The results may indicate that systemic T cell exhaustion, including of tumor-infiltrating lymphocytes and circulating CAR T cells, associates with a lack of durable response to cell therapy.

### Evolution of tumor immune contexture at relapse

**Extended Data Fig. 8:**
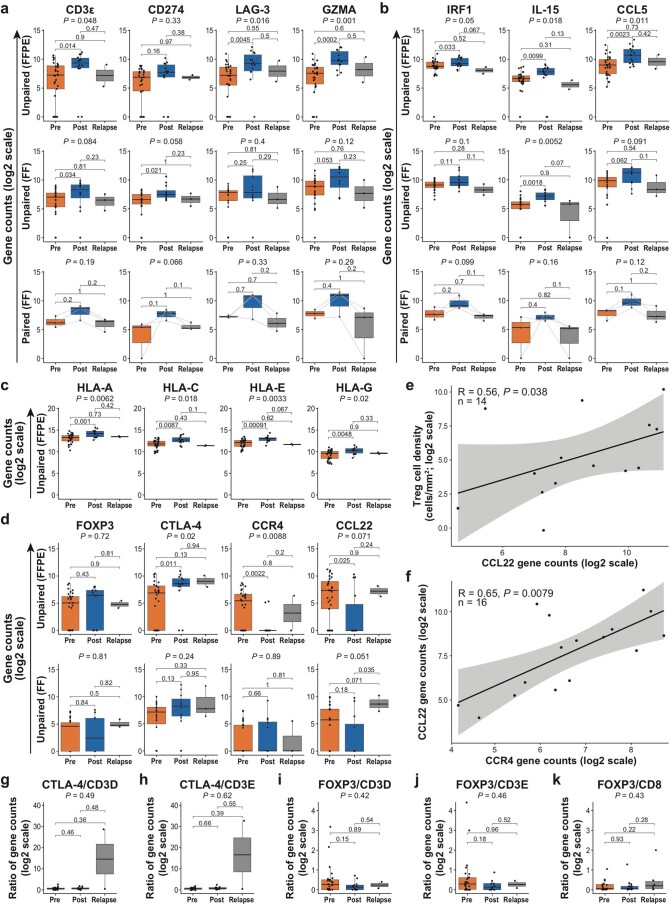
Evolution of immune gene expression in tumour biopsies of ZUMA-1 patients who relapsed. Gene expression was measured with PanCancer Immune Profiling panel in tumour biopsies from unpaired or paired samples (as indicated) of Zuma 1 patients from subset 1 (n=39; 19 unpaired FFPE; 23 unpaired FF; 3 paired FF), subset 2 (n=22; 14 unpaired FFPE; 12 unpaired FF; 3 paired FF), and subset 3 (n=4; 2 unpaired FFPE; 3 unpaired FF; 3 paired FF,). a-c, Gene expression related to anti-tumour immune response. a, Cytotoxic T-cell–related genes; b, cytokine and IFN-related genes; c, MHC class I–related genes. P values were derived with a Kruskal-Wallis test. d-i, Gene expression related to immunosuppressive response. d, Comparative gene expression of FoxP3, CTLA-4, CCR4, and CCL22 genes (Kruskal-Wallis test; P values as indicated) in biopsies from subset 1 (n = 19), subset 2 (n = 14), and subset 3 (n = 2); e-i, Ratios of gene expression for CTLA-4 with (e) CD3D or (f) CD3E (statistical significance of the spearman coefficient level (two-sided P value) embedded in panels; grey ribbons represent the 95% Confidence Interval of the regression line) and of FoxP3 with (g) CD3D, (h) CD3E, or (i) CD8A (Kruskal-Wallis test, P values as indicated). j,k, Correlations between gene expression of CCL22 and cell density of Treg measured by Immunoscore TCE panel (j) or gene expression of CCR4 (k) in tumour biopsies from Zuma 1 subset 1 pts (n = 14 and n = 16, respectively),two-sided Wilcoxon tests. axi-cel, axicabtagene ciloleucel; CCL, chemokine ligand; CCR, chemokine receptor; CTLA-4, cytotoxic T lymphocyte–associated protein 4; FOXP3, forkhead box P3; GZMA, granzyme A; HLA, human leucocyte antigen; IL, interleukin; IRF1, interferon regulatory factor 1; LAG-3, lymphocyte-activation gene 3; MHC, major histocompatibility complex; TCE, T-cell Exhaustion; TME, tumour microenvironment; Treg, regulatory T-cell.

**Extended Data Fig. 9:**
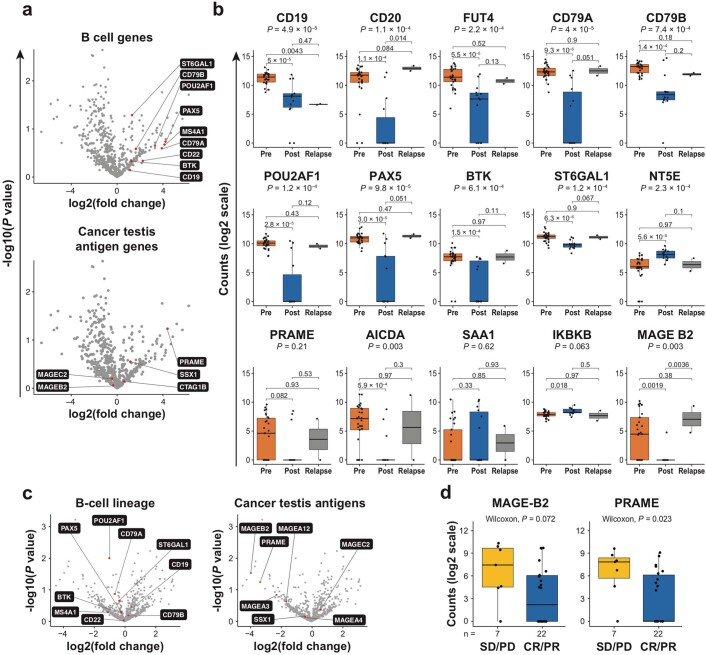
B-cell lineage and CTA gene expression in tumour biopsies of ZUMA-1 patients. Gene expression was measured with PanCancer Immune Profiling panel in Zuma 1 (a,b) tumour biopsies from subsets 1, 2, and 3, and (c,d) in pretreatment tumour biopsies from patients with CR (n=22 total [20 CR, 2 PR]) vs. nonCR (n=7 total [4 SD, 3 PD]) a, Volcano plots of B-cell lineage (top panel) and CTA (bottom panel) gene expression from subset 2 (n = 12) and subset 3 (n = 3). The plot was constructed using log2(fold change) and –log10(P value) for all genes analysed by PanCancer Immune Profiling panel. Red dots represent the top differentially expressed genes with P<0.01 (two-sided t.test without adjustment). b, Evolution of B-cell and CTA gene expression across FFPE biopsies from subsets 1 (n=19), 2 (n=14), and 3 (n=2). c, Volcano plots of B-cell lineage (left) and CTA-related (right) gene expression in pretreatment tumour biopsies of patients with CR vs. nonCR outcomes. P values were derived with a Kruskal-Wallis test. d, Gene expression of CTA-related genes (MAGE-B2 and PRAME) in pretreatment tumour biopsies of patients with CR/PR vs. SD/PD outcomes. P values were derived with two-sided Wilcoxon test.AICDA, activation-induced cytidine deaminase; axi-cel, axicabtagene ciloleucel; BTK, Bruton tyrosine kinase; CR, complete response; CTA, cancer testis antigen; FFPE, formalin-fixed paraffin-embedded; IKBKB, inhibitor of nuclear factor kappa B kinase subunit B; ; IS21, Immunosign21; MAGE, melanoma-associated antigen; PAX5, paired box protein 5; PD, progressive disease; POU2AF1, POU domain class 2-associating factor 1; PR, partial response; PRAME, preferentially expressed antigen in melanoma; SAA1, serum amyloid A1; SD, stable disease; TME, tumour microenvironment.

We interrogated TME dynamic changes of axi-cel–treated patients developing secondary treatment resistance after 6 months^5^. As our dataset of patients who relapsed after achieving a clinical response to axi-cel was limited, analyses were performed in matched FF and FFPE biopsies and in paired samples (*n* = 7, all patients with longitudinal samples and three patients with both FF and FFPE biopsies). The TME at relapse evolved toward a global reduction in expression of T cell–related and IC genes, cytokine and IFN-related genes and major histocompatibility complex (MHC) class I–related genes compared to early post-treatment TME (Extended Data Fig. 8a–c). The results were consistent across FF (upper rows), FFPE (middle rows) and paired (lower rows) biopsies (Extended Data Fig. 8a,b). Concurrently, an increase in immune counter-regulatory and tumor-associated markers was observed at relapse, including genes involved in immune suppression, CTLA-4, CCR4 (ref. ^38^) and CCL22 (ref. ^39^) (Extended Data Fig. 8d; upper row, FFPE biopsies, and lower row, FF biopsies). TME composition at relapse significantly associated with an increase of CTLA4 gene counts normalized to tumor-infiltrating CD3 gene counts (Extended Data Fig. 8e,f) but not of FoxP3 gene (Extended Data Fig. 8g,i). Consistent with these findings, CCL22 gene counts correlated with Treg cell density (Extended Data Fig. 8j) and with CCR4 gene counts in subset 1 (Extended Data Fig. 8k). Unsurprisingly, B cell lineage and CTA gene expression also significantly increased at relapse (Extended Data Fig. 9).

### Pre-treatment immune contexture associated with survival

**Fig. 4:**
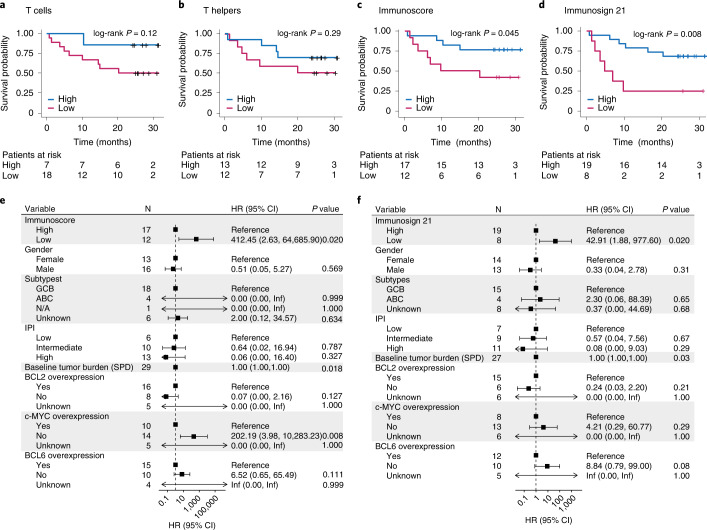
Pre-treatment tumor immune contexture associated with survival probability of patients in ZUMA-1. **a**–**d**, Overall survival of patients in ZUMA-1 as a function of Tc (**a**) or Th (**b**) cell infiltration in pre-treatment TME, Immunoscore index (**c**) or Immunosign 21 score (**d**). The *P* values were generated by using an unweighted log-rank test with survminer package in R. **e**,**f**, Survival hazard ratios from multivariate analyses of ZUMA-1 patient clinical characteristics and pre-treatment tumor biopsies analyzed for Immunoscore (*n* = 29) (**e**) and Immunosign 21 (*n* = 27 (**f**). TB analysis was performed as continuous variable. Cox multivariate regression was used to calculate hazard ratio statistical significance. *P* values are two-sided. CI, confidence interval; HR, hazard ratio; Inf, infinity; N/A, no available molecular data to determine subtype; PD, progressive disease; PR, partial response; SD, stable disease; SPD, sum of the products of diameters.

We next addressed whether pre-treatment T cell infiltration in the TME correlated with efficacy and survival of patients in ZUMA-1. Although T cell–related genes and T cell densities only modestly trended with axi-cel response (Tc density, *P* = 0.12, and Th density, *P* = 0.29; Fig. 4a,b), the pre-defined Immunoscore index and Immunosign 21 significantly correlated with overall survival (Immunoscore*, P* = 0.045, and Immunosign 21, *P* = 0.008; Fig. 4c,d).

Multivariate analyses with clinical and molecular features further confirmed that survival hazard ratios significantly associated with Immunoscore and Immunosign 21 (Fig. 4e,f; *P* = 0.020) as well as TB (Fig. 4e,f; *P* = 0.018/0.03), consistent with previously published data^34^. These results emphasized the reliability of Immunoscore and Immunosign 21 scoring of T cell tumor infiltration across datasets, but we cannot exclude some inter-dependency between these two covariates, which will need further exploration using larger datasets.

**Fig. 5:**
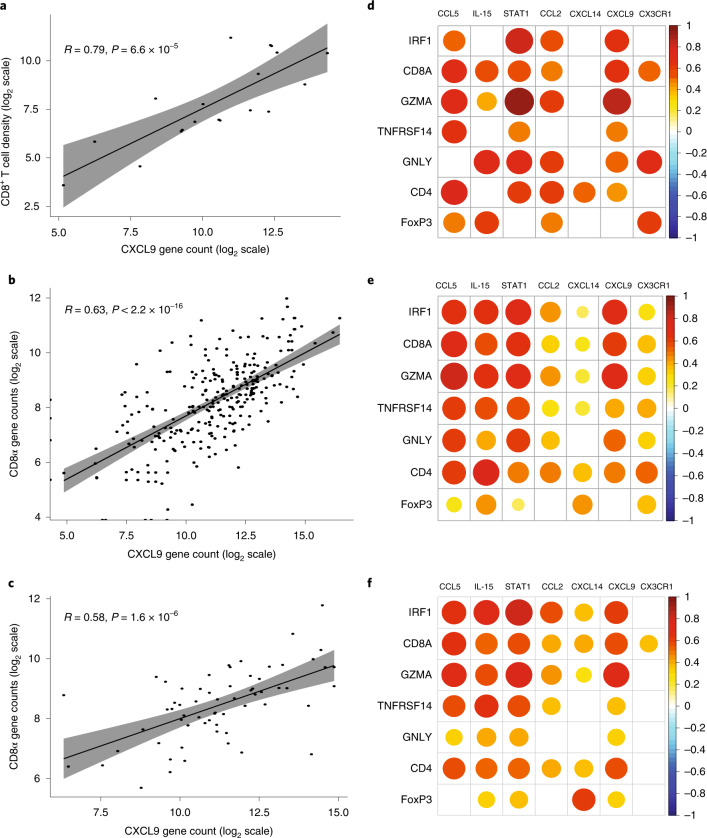
T cell subsets in pre-treatment tumor biopsies associated with myeloid-secreted chemokines. **a**–**c**, Correlation between CXCL9 gene expression in pre-treatment tumor biopsies and CD8^+^ T cell tumor infiltrate measured by Immunoscore TCE panel (**a**) or CD8A gene expression (**b**,**c**) in patients in ZUMA-1 (subset 1; *n* = 19) (**a**), second-line patients with DLBCL (*n* = 252) (**b**) and treatment-naive patients with DLBCL (*n* = 67) (**c**). Gene expression was measured by PanCancer Immune panel for **a** and **c** and Immuno-Oncology 360 panel for **b**. **d**–**f**, Correlation matrix for myeloid-secreted and/or T cell–produced cytokines and chemokines (horizontal axis) with T cell subset–related genes (vertical axis) in pre-treatment tumor biopsies from ZUMA-1 (**d**), second-line (**e**) and treatment-naive patients with DLBCL (**f**). The scale bar (−1 to 1) represents the *R* value. The gray ribbons represent the 95% confidence interval of the regression line. Statistical significance of the Spearman coefficient level (two-sided *P* value as shown) was calculated. CCL, chemokine ligand; CXCL, chemokine C-X-C motif ligand; FoxP3, forkhead box P3; GNLY, granulysin; GZMA, granzyme A; IL, interleukin; IRF1, interferon regulatory factor 1; PD, progressive disease; PR, partial response; SD, stable disease; STAT, signal transducer and activator of transcription; NFRSF14, tumour necrosis factor receptor superfamily member 14.

**Extended Data Fig. 10:**
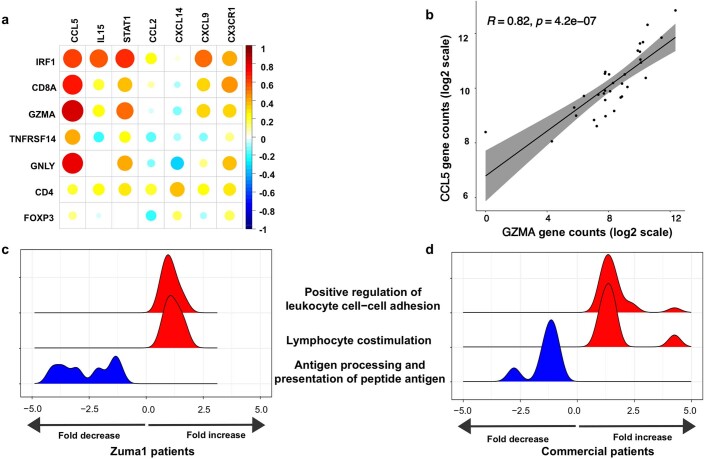
Correlative studies in Zuma 1 and commercial axi-cel patient datasets. a,b,d. Pretreatment tumour biopsies from commercial patients (r/r DLBCL) were analysed with IO360 NanoString panel (n=33; 20 CR and 10 non-CR; 3 samples failed nanostring QC). a, Correlation matrix for myeloid-secreted and/or T-cell–produced cytokine/chemokines (horizontal axis) with T-cell subset–related genes (vertical axis) (Spearman R). The scale bar (-1 to 1) represents the R value. b, Gene expression correlation of CCL5 chemokine and cytotoxic T-cells–produced serine protease GZMA. Statistical significance of the spearman coefficient level (two-sided P value) in the panels. The grey ribbon represents the 95% Confidence Interval of the regression line. c,d. Comparison of regulation of three functional pathways (“Positive regulation of leukocyte cell-cell adhesion", "Lymphocyte costimulation", and "Antigen processing and peptide antigen presentation") in pretreatment, CR vs. non-CR, tumour biopsies from Zuma 1 (c, 20 CR, 9 non-CR) and commercial (d, 20 CR, 10 non-CR) patients. Functional pathways with statistically significant p values < 0.005 were selected and GSEA adjusted p.values were calculated with Benjamini-Hochberg method. Adjusted two-sided p.values of less than 0.5 in at least one of the two panels are shown. axi-cel, axicabtagene ciloleucel; CCL, chemokine ligand; CXCL, chemokine C-X-C motif ligand; DLBCL, diffuse large B-cell lymphoma; CR, complete response; non-CR, non-complete response; FoxP3, forkhead box P3; GNLY, granulysin; GZMA, granzyme A; IL, interleukin; IRF1, interferon regulatory factor 1; QC, quality control; r/r, relapse, refractory; STAT, signal transducer and activator of transcription; TNFRSF14, tumour necrosis factor receptor.

We next interrogated TME gene expression of chemokines, cytokines and their receptors for possible association with T cell genes and T cell density. We analyzed the association between T cell subsets and chemokine and cytokine expression in pre-treatment TME across four independent datasets. Available transcriptomics data from an ongoing, second-line DLBCL, CAR T cell interventional study^25^, as well as published results from commercial patients treated at Moffitt Cancer Center^26^, were added to ZUMA-1 and treatment-naive DLBCL datasets already described. The dataset from the ongoing, second-line DLBLC clinical trial could not be used to validate associations with clinical outcomes. Chemokines secreted only by myeloid cells (CXCL9 and CXCL14) or by both myeloid and T cells (CCL5 and its receptor CCR5), as well as STAT1 gene expression, were directly associated with T cell density in all four datasets (Fig. 5 and Extended Data Fig. 10). T cell genes (CD3δ, CD8 and CD4) also correlated with genes encoding cytokines important for T cell expansion, stemness, viability and differentiation, such as IL-21 Th cells or IL-7, IL-18 and IL-15 produced by stromal or myeloid cells^40,41^ (Supplementary Table 10).

We next sought to validate biological associations seen in the ZUMA-1 cohort using other patient independent datasets extracted from a second-line DLBCL clinical trial^25^ and a commercial axi-cel dataset. In second-line DLBCL, IL-15 and IL-18 correlated with CD3ε gene expression (Spearman *P* < 1 × 10^−16^ and *P* = 6.6 × 10^−16^, respectively). These results were also confirmed in 33 commercial patients. Extended Data Fig. 10a,b shows a robust correlation between activated T cell markers (GZMA and GNLY) and chemokines (CCL5)^26^ in the commercial axi-cel datasets. Finally, the analysis of the main immune pathways associated with CR compared to non-CR in two independent datasets (ZUMA-1 and commercial axi-cel patients) revealed that, in each patient group, lymphocyte co-stimulation and leukocyte cell–cell adhesion were associated with increased fold change, whereas antigen processing and presentation of peptide antigen genes related to M2 macrophages and myeloid activation (CD206, CD36, CD74, HLA-DM, HLA-DQ, HLA-DRA, HLA-E and TREM2) were associated with a decrease in fold change (CR versus non-CR; Extended Data Fig. 10c,d).

Altogether, these data linked key pre-treatment tumor immune characteristics, including chemokines and γ-chain receptor cytokine expression, to a T cell–involved immune contexture and may indicate actionable functional pathways to sensitize the TME to CAR T cell therapy.

### Pre-treatment TME T cell subsets associated with survival

**Fig. 6:**
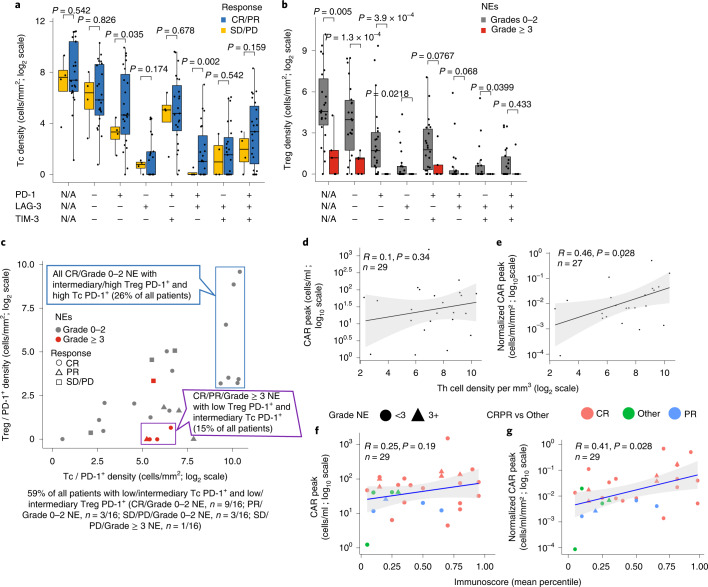
Cell density of T cell subsets in pre-treatment tumor biopsies associated with axi-cel efficacy, NEs and CAR T cell expansion. T cell subset densities were measured by Immunoscore TCE panel in ZUMA-1 tumor biopsies (*n* = 27) and plotted as a function of clinical response to axi-cel (CR/PR versus SD/PD; 19 CR, four PR and four SD/PD) or NE grade (grades 0–2, *n* = 22, versus grade ≥3, *n* = 5) and IC expression (PD-1, LAG-3 and TIM3). **a**, Tc density versus clinical response and IC expression. **b**, Treg density versus NE grade and IC expression. N/A = not applicable, regardless of PD-1, LAG3, TIM-3 expression (**a**,**b**). **c**, Correlation of clinical outcomes and NE grades with densities of tumor-infiltrating Tc and Treg cells. Two-sided exact Fisher test *P* value of responders with NE grade ≥3 and low Treg density (purple box) versus all other patients = 5.698 × 10^−5^; two-sided exact Fisher test *P* value of complete responders with NE grades 0–2 and high Treg density (blue box) versus all other patients = 0.02. **d**,**e**, Correlations of non-activated Th density measured by Immunoscore TCE panel and peak CAR T cells without (*n* = 23) (**d**) or with (*n* = 19) (**e**) normalization to pre-treatment TB. **f**,**g**, Correlations of Immunoscore and peak CAR T cells without (*n* = 29) (**f**) or with (*n* = 27) (**g**) normalization to pre-treatment TB. The gray ribbons (**d**–**g**) represent the 95% confidence interval of the regression line. Statistical significance of the Spearman coefficient level (two-sided *P* value) as shown was calculated. LAG-3, lymphocyte activation gene 3; PD, progressive disease; PD-1, programmed cell death protein 1; PR, partial response; SD, stable disease; TIM-3, T cell immunoglobulin and mucin domain 3.

To identify the cell subsets associated with axi-cel outcomes in the patients in ZUMA-1, we measured the T cell density in pre-treatment TME with Immunoscore TCE and the expression levels of B cell lineage genes. The density of CD8^+^ T cells with activated phenotype (PD-1^+^ checkpoint and LAG3^+/−^TIM3^−^) was most significantly associated with OR, contrasting with other CD8^+^ T cell subsets, including non-activated (no checkpoint expression) or exhausted (three checkpoints (PD-1^+^LAG-3^+^TIM-3^+^)) T cells (Fig. 6a). But the expression levels of B cell lineage genes, including CD19 (CAR target), CD79b, CD22 and PAX5, were similar across outcome groups (Extended Data Fig. 9c), whereas the expression of some CTA genes (PRAME and MAGE-B2 family members) was more elevated in non-responders (Extended Data Fig. 9d). The cell density of various myeloid subsets, including CD11b^+^CD15^−^CD14^+^ monocytes and CD68^+^ macrophages, was greater than the T cell density in pre-treatment TME (Supplementary Table 11), but the overall myeloid subset density was not significantly associated with clinical outcomes (Supplementary Fig. 2)

In contrast, Treg density (CD3^+^CD8^−^FoxP3^+^) was markedly reduced in patients who developed high-grade (grade ≥3) NEs after axi-cel, independently of checkpoint expression (Fig. 6b; *P* < 0.0001; Supplementary Fig. 3a). Unexpectedly, Treg density associated positively with TME features favoring axi-cel clinical response, including high CD8^+^PD-1^+^ T cell density (Supplementary Fig. 3b). Patients with high density of CD8^+^PD-1^+^ T cells and intermediary/high density of PD-1^+^ Tregs (≈1/4 of all tested patients) achieved CR without serious NEs after axi-cel. Most patients (*n* = 4/5) with low density of Tregs yet measurable CD8^+^PD-1^+^ T cells in pre-treatment TME developed grade ≥3 NEs after axi-cel (Fig. 6c, purple box; Fisher *P* = 5.7 × 10^−5^). Furthermore, pre-treatment tumor-infiltrating non-activated Th cells positively associated with peak CAR T cell levels, with or without normalization to TB (Fig. 6de), and pre-treatment Immunoscore significantly associated with normalized peak CAR T cell levels (*P* = 0.028; Fig. 6g). These data suggest that pre-treatment subsets of tumor-infiltrating lymphocytes, such as activated CD8^+^ T cells and Treg cells, associate differentially with efficacy and neurotoxicity after axi-cel.

## Discussion

Recent findings suggest that CAR T cell therapy may have curative potential in select patients with non-Hodgkin’s lymphoma^42^, but existing prognostic subgroups do not predict response to axi-cel^8^. As preclinical models typically fail to mimic accurately the complex mechanisms of action of immunotherapy, reverse translational studies were undertaken to identify tumor-related features associated with outcomes to CAR T cell intervention. We performed comprehensive gene expression analyses on more than 400 patients with LBCL treated with axi-cel (ZUMA-1, third-line, r/r LBCL; third-line, relapse LBCL commercial axi-cel patients; patients with DLBCL enrolled in an ongoing, second-line, CAR T cell study;^25^ and treatment-naive patients with DLBCL) as well as multiplex spatial tissue analysis on 118 ZUMA-1 and treatment-naive patients with DLBCL. Clinical outcome data were available only for the ZUMA-1 patient datasets; the second-line and treatment-naive patient datasets served as validation for biological associations. We discovered that tumor immune contexture was associated with, and potentially a major determinant of, axi-cel clinical efficacy in patients in ZUMA-1. The key findings of this study are: (1) rapid change of immune TME features after axi-cel; (2) CR to CAR T cell treatment associated with pre-treatment TME rich in cytokines and chemokines that favors T cell involvement; (3) high density of PD-1^+^LAG-3^+/–^ T cells in pre-treatment TME associated with OR; (4) low density of Tregs in pre-treatment TME associated with grade ≥3 neurotoxicity; (5) overall survival associated with high Immunoscore and Immunosign 21 in pre-treatment TME; and (6) the higher the T cell exhaustion in post-treatment TME, the lower the circulating CAR T cell levels, a key correlate of durable response^34^.

This study demonstrated that TME gene signature evolution occurs quickly after axi-cel treatment, whereby responders show rapid upregulation of the T cell–related signature and downregulation of B cell tumor-related markers, defining a dynamic pattern that separates responders from non-responders within 2 weeks post-treatment. The early TME gene expression changes that were observed may serve as a pharmacodynamic marker of response. Patients with low pre-treatment TB and high T cell involvement showed higher likelihood of achieving CR, but, when response was achieved, the TME evolved similarly in small versus large TB. The pre-specified Immunoscore and Immunosign 21 indexes—pertaining to T cell activity and related immune programs—were associated with overall survival, with clinical follow-up exceeding 1 year. The density of activated CD8^+^PD-1^+^LAG-3^+/−^TIM-3^−^ T cells was the TME feature most associated with clinical efficacy. In addition, pre-treatment TME density of CD3^+^CD8^−^FoxP3^+^ Tregs and related features associated with lower-grade axi-cel–related neurotoxicity without a notable association with efficacy, suggesting overall better T cell infiltration and/or a beneficial effect of TME-infiltrating Tregs on the therapeutic index in this setting possibly related to the essential role of Tregs in normal tissue protection^43,44^. At relapse, the TME acquired an immune-detrimental contexture with decreased T cell–related signature and increased tumor-associated and immune counter-regulatory markers^45^, including CCR4 and CCL22. Finally, pre-treatment tumor immune contexture associated with T cell presence and activity in TME through chemokines (for example, CXCL9 and CXCL14) and cytokines (for example, IL-15, IL-7, IL-18 and IL-21) produced locally (Fig. 5, Extended Data Fig. 10, Supplementary Fig. 4 and Supplementary Table 10), which supports the hypothesis that stromal production of T cell–attractive chemokines and γ-chain receptor cytokines may promote a T cell–involved TME generally favorable for CAR T cell activity.

The positive association between high density of CD8^+^PD-1^+^ T cells in pre-treatment TME and axi-cel response mirrors previous findings in solid tumors describing response to IC blockade^10,15,22,46^ and expands the paradigm that immunotherapy^47^, including CAR T cell therapy, is favored by an immune-involved TME^48,49^. Overall survival also was positively correlated with high Immunoscore index and Immunosign 21 score. The immune contexture, as measured by the pre-specified gene expression panel Immunosign 21, has been linked to prediction of response to multiple immunotherapies in multiple cancer types^22^. It would, thus, be of interest to also evaluate pre-treatment Immunosign 21 before CAR T cell therapy for solid tumors.

Furthermore, axi-cel expansion relative to TB, a known robust correlate of durable efficacy^5,34^, was positively associated with a T cell–involved TME at baseline and negatively associated with exhausted (TOX^+^) tumor-infiltrating T-cells after axi-cel, providing a key rationale to link pre-treatment and post-treatment tumor immune contexture and axi-cel performance. In brief, patients with lower pre-treatment TB, higher density of activated CD8^+^ T cells and measurable Tregs within the TME may have a more favorable clinical evolution in terms of efficacy and toxicity after axi-cel, and these parameters could be included in the design of future clinical trials.

This analysis—directed at comparing patients by best response to axi-cel—has not identified a clear association between pre-treatment myeloid cell signature in the TME and clinical outcomes or between COO classification and the response rate to axi-cel in patients with r/r LBCL. Other studies recently identified a link between specific myeloid cell signatures and CAR T cell treatment-related toxicities^50,51^ and durable response, respectively^26^. The study by Jain et al.^26^ investigated a distinct question—namely, the effect of myeloid cells (M-MDSCs) and associated IFN signalling on the process of relapse in patients who responded to axi-cel, indicating that a dysregulated myeloid signature may be mechanistically involved in the process. Immunoscore index and Immunosign 21 were identified as significant predictors of overall survival after CAR T cell treatment (Fig. 4), and the latter was correlated with T cell subset densities (*P* < 0.005) but not with macrophages or mononuclear subsets (*P* = 0.69 and *P* = 0.27, respectively; Extended Data Fig. 6b) in patients in ZUMA-1. Interestingly, in treatment-naive and second-line patients with DLBCL, Immunosign 21 did correlate with myeloid subsets (macrophage density, *P* = 0.0077, and CD68 gene expression, *P* = 5.5 × 10^−15^, respectively; Extended Data Fig. 6c,d), in addition to CD8 T cells. Furthermore, lower Immunoscore and Immunosign 21 associated with ABC subtype in treatment-naive patients, but COO did not correlate with the overall survival of the patients in ZUMA-1 reported in this manuscript (Figs. 3 and 4). These observations suggest that molecular attributes of DLBCL subtypes initially influence the TME composition but are not associated with CAR T cell response. In contrast, the immune infiltrate, as well as TB, was associated with response to CAR T cells, and multiple lines of chemotherapy may later modify the composition of the immune infiltrate.

In addition to yielding predictive markers, these findings suggest strategies to overcome treatment resistance in patients with immune-detrimental TME through local or systemic provision of T cell chemokines, γ-chain receptor cytokines or IFN program-stimulating factors, using T cell engineering or combinatorial approaches^26,47,52,53^. These approaches may include epigenetic modulators aimed at restoring dysregulated gene expression manifesting through elevated CTAs^28,54,55^ or TOX, the central regulator of exhausted CD88^+^ T cells^56^, which were found to be negatively associated with clinical response to axi-cel. Supporting the latter, current CAR T cell generation has a propensity for premature antigen-induced cell exhaustion and death owing to supraphysiological signaling^57^.

Our study has several limitations. The only sample dataset with linked clinical outcomes was the ZUMA-1 patient dataset, and the sample size for relapse and paired subsets was small. Also, CAR T cell probes were not included in the study because CAR levels in the TME were unexpectedly low in biopsies performed 2 weeks after axi-cel. Earlier immunohistochemical and in situ data have shown that CAR T cells make up only a very small percentage of intratumoral T cells 5 days or more after axi-cel infusion^48^. In addition, in two prior studies of tumoral transcriptomics predicting clinical outcomes to CAR T therapy^58,59^, clinical benefit was associated with tumor expression of death receptors, but the content of the transcriptomics panels employed in this study did not allow us to investigate other possible tumor cell intrinsic factors, such expression of death receptors. In two prior studies of tumoral transcriptomics predicting clinical outcomes to CAR T therapy^58,59^, both showed the benefit of tumoral expression of death receptors. Finally, the broad deployment of immune responses after CAR T infusion contrasts with the unexpected low frequency of detectable autologous CAR T cells in the TME. Reduced CAR expression on the membrane surface after antigen stimulation could occur through several mechanisms, hampering detection by IHC-based and RNA-based methods. Alternative approaches to detection of CAR T cells in the TME are warranted before translating the results of this study to other cell therapy modalities.

In conclusion, this study advances understanding of axi-cel mechanism of action, linking its performance (that is, CAR T cell efficacy, toxicity and patient survival) to tumor immune contexture pre-treatment and post-treatment (Supplementary Fig. 4). This study also highlights a third mechanism of resistance to axi-cel in third-line DLBCL, in addition to product T cell fitness^34^ and target-related evasion^60^, that is related to the tumor immune microenvironment. Although our sample size was limited, these results, pending validation, suggest that immune-based therapies with curative potential, such as axi-cel, should be considered in earlier lines of therapy where a larger percentage of patients have more favorable TME features and lower TB, to potentially maximize clinical benefit and curative potential. Given the practical implications, further TME signature optimization and validation in larger studies is warranted in LBCL and in other tumor types for which T cell therapies are being developed.

## Methods

### Patient sample collection and preparation

The study protocol for the single-arm, multicenter, registrational ZUMA-1 study of axi-cel in patients with relapsed LBCL was previously described^5,24^. Each study site’s institutional review board reviewed and approved the study protocol and amendments, and all patients provided written informed consent. The study was done according to the International Conference on Harmonisation Good Clinical Practice guidelines. Patients in the ZUMA-1 study did not receive compensation for their participation. Eligible patients had histologically confirmed LBCL and refractory disease, defined as progressive or stable disease as best response to most recent chemotherapy regimen or disease progression or relapse within 12 months after autologous stem cell transplantation^5,24^. Patients received axi-cel at a target dose of 2.0 × 10^6^ CAR T cells per kilogram^5,24^. Tumor biopsy specimens from patients in ZUMA-1 (Extended Data Fig. 1) were collected at baseline (before conditioning chemotherapy (pre-lymphodepletion)) and after axi-cel infusion. The largest group of samples (40%) was from lymph nodes, but no significant differences in the TME composition between tumor originating from lymph nodes versus other origins were found by principal component analysis (PCA) of all gene expression for FFPE baseline samples (Extended Data Fig. 1b). Patients with samples collected before axi-cel infusion are referred to throughout the manuscript as subset 1, early after CAR T cell infusion (days 7–14) as subset 2 and later at relapse as subset 3 (Extended Data Fig. 1c). Of note, not all patient samples are on all figures. Inclusion of samples was based on the following. (1) In addition to the informed consent statement for trial participation and primary analysis, consent for exploratory analysis was required, which reduces the amount from the total number of patients enrolled in the ZUMA-1 phase 2 pivotal trial. (2) Remaining sample available for testing. (3) Samples that passed quality control (QC) for any given platform, as described below. After sample quality was controlled for, a total of 135 baseline and post-treatment biopsy specimens from 51 patients in ZUMA-1 with LBCL were analyzed for this study. The patient characteristics and clinical outcomes analyzed in this study were compared to those of the overall ZUMA-1 population. No significant differences were found (*t*-test *P* values for age = 1; *P* value for sex = 0.318; *P* value for best response = 0.175; *P* value for disease type comparison = 0.664; *P* value for International Prognostic Index (IPI) = 0.846; *P* value for more than three prior lines of therapy = 0.664; *P* value for CAR T cell levels = 0.854). All post-treatment biopsy specimens were acquired between days 7 and 14, except for a specimen collected at week 4 from a patient with stable disease. Subset 1 (baseline) includes 46 patients, with nine patients with FF biopsies, 19 patients with FFPE biopsies and 18 patients with both types of biopsies. Subset 2 (post-treatment) includes 28 patients, with nine patients with FF biopsies, ten patients with FFPE biopsies and nine patients with both. Subset 3 (relapse) includes seven patients, with one patient with FF biopsies, four patients with FFPE biopsies and two patients with both (Extended Data Fig. 1d). The patient characteristics and clinical outcomes described in this study were consistent with those observed in the overall ZUMA-1 cohort (Supplementary Table 1). Best response was evaluated within 2 years of axi-cel infusion for patients enrolled in ZUMA-1 phases 1 and 2 (cohorts 1 and 2) and within 6 months of axi-cel infusion for patients enrolled in ZUMA-1 phase 2 (cohort 3). NEs in ZUMA-1 were graded according to Common Terminology Criteria for Adverse Events, version 4 (refs. ^5,24^).

Three independent datasets were also included in this study (Supplementary Table 3)—namely, 67 biopsy specimens from treatment-naive patients with DLBCL (collected at time of diagnosis), 252 biopsy specimens from patients with r/r DLBCL in an ongoing open-label, second-line, interventional study (NCT03391466; study is still blinded to outcomes) and 33 biopsy specimens from axi-cel commercial patients treated at Moffitt Cancer Center^26^. Baseline tumor samples were analyzed by targeted transcriptomics (Immuno-Oncology 360) for the 252 second-line patients and 33 axi-cel commercial patients treated at Moffitt Cancer Center.

### Gene expression profiling

Gene expression profiling was performed using three gene panels (Supplementary Table 4) on 89 biopsy specimens from ZUMA-1 patient subsets 1, 2 and 3. Twenty-four patients had longitudinal biopsy specimens (baseline/post-infusion, *n* = 17; baseline/post-infusion/relapse, *n* = 6; baseline/relapse, *n* = 1). Clinical research tests, including Immunoscore TL (consensus Immunoscore assay CD3 and CD8), Immunoscore TCE (CD3, CD8, FoxP3, PD-1, LAG-3 and TIM-3), Immunoscore TCE^+^ (CD3, CD8, FoxP3, PD-1, LAG-3, TIM-3, TOX ND and EZH2), Immunoscore SC (CD11b, CD14, CD15, CD68, S100A9 and LOX-1) and Immunosign 21 (Supplementary Table 4), were performed in a Clinical Laboratory Improvement Amendments–certified laboratory (HalioDx). For ZUMA-1 pre/post–axi-cel comparisons, biopsy specimens were analyzed using the PanCancer Immune Profiling Panel. For pre-treatment (baseline) analyses, biopsy specimens were analyzed using the PanCancer Immune + Immunosign Gene Panel. Slide sets were prepared from each FFPE block by cutting ten consecutive 4-µm sections, which were further immobilized on Superfrost Plus slides. One slide was used for hematoxylin and eosin (H&E) staining; two consecutive slides were used for Immunoscore TL (automatic staining CD3/CD8, BenchMark XT); and four or five consecutive slides were used for RNA extraction and NanoString profiling. Slides were de-identified and tracked using a unique number for each pre-analytical step, and two workflows per patient were performed

### Sample QC

QC at various steps was implemented to remove samples or data that failed to meet requirements. Incoming FFPE blocks were visually inspected to assess block quality or to determine whether sufficient material remained. Tissue sections were assessed for defects in section quality. H&E stains were assessed for image quality and necrosis by a pathologist. Slides with more than 95% necrotic tissues were not stained. Because most DLBCL biopsy specimens lacked an identifiable invasive margin, the percentage of tumor content was not part of the initial tissue selection for staining. After multiplex IHC panels, technical criteria—including tissue staining QC of tissue sections, image QC for blurry areas or artifacts and digital pathology QC (specificity, intensity, detection sensitivity and detection of tumor areas)—were used to select study samples. For RNA sequencing, samples that did not meet input requirements or had extensive degradation were eliminated. Suitable samples were then assessed for library preparation metrics, sequencing QC (Q30 > 70%) and analysis QC (10 million assigned reads obtained). For NanoString analyses, samples that did not have sufficient input and any samples flagged as failing raw data QC or normalization QC by NCounter software during analysis were removed. The starting number of samples and final number of samples that passed all QC metrics and were, therefore, included in the various analyses are stated below for the respective assays.

### Antibody validation

The CD3 (HDX1) and CD8 (HDX2) primary antibody performances have been established and validated for the Immunoscore colon cancer test (CLIA and CE-IVD marked test) and are produced by Veracyte. Commercial antibody characteristics are listed in Supplementary Table 12 and include CD3 (1 μg ml^−1^)^61^, CD8 (1 μg ml^−1^)^61^, PD1 (1/8,000, 0.125 μg ml^−1^; https://cdn.origene.com/datasheet/um800091.pdf)^62^, LAG3 (1/400, 1.95 μg ml^−1^; https://www.cellsignal.com/products/primary-antibodies/lag3-d2g4o-xp-rabbit-mab/15372)^63^, TIM3 (1/400, 0.04 μg ml^−1^; https://www.cellsignal.com/products/primary-antibodies/tim-3-d5d5r-xp-rabbit-mab/45208)^32^, FoxP3 (1/100, 5 μg ml^−1^; https://www.thermofisher.com/antibody/product/FOXP3-Antibody-clone-236A-E7-Monoclonal/14-4777-82)^64^, TOX (1/100, 10 μg ml^−1^; https://www.abcam.com/tox-antibody-nan448b-ab237009.html)^65^, EZH2 (1/800, 0.53 μg ml^−1^; https://www.cellsignal.com/products/primary-antibodies/ezh2-d2c9-xp-rabbit-mab/5246)^66^, LOX1 (1/800, 1.25 μg ml^−1^; https://www.merckmillipore.com/FR/fr/product/Anti-LOX-1-clone-9E12.1,MM_NF-MABS186), CD68 (1/1,000, 0.7 μg ml^−1^; https://www.abcam.com/cd68-antibody-epr20545-ab213363.html)^67^, CD11b (1/200, 0.5 μg ml^−1^; https://www.cellsignal.com/products/primary-antibodies/cd11b-itgam-d6x1n-rabbit-mab/49420)^68^, CD14 (1/50, 0.07 μg ml^−1^; https://www.cellmarque.com/antibodies/CM/2066/CD14_EPR3653)^69^, CD15 (1/100, 5 μg ml^−1^; https://www.bdbiosciences.com/en-us/search-results?searchKey=555400)^70^ and S100A9 (1/16,666, 0.06 μg ml^−1^; https://cdn.origene.com/datasheet/um800066.pdf). The primary antibodies were selected to cross-react on human tissues and were recommended by the suppliers for IHC on FFPE tissues. Supporting literature regarding antibody specificity is found in the suppliers’ documentation. For Immunoscore testing, the specificity of the selected primary antibodies was verified using human tonsil as positive tissue for targeted immune biomarkers. Simplex IHC protocol was further optimized for each biomarker using DLBCL FFPE samples before the multiplex IHC.

### Targeted transcriptomics analysis and Immunosign scores

RNA was extracted from frozen or fixed biopsy specimens using a QIAGEN RNeasy kit or QIAGEN RNeasy FFPE extraction kit, respectively. Annotations from the pathologist performing the H&E staining were used to guide removal of normal tissue from the slides by microdissection before RNA extraction, which occurred after tissue deparaffinization and lysis. Each RNA extraction was independently quantified (NanoDrop) and qualified (Agilent Bioanalyzer). Degradation assessment was quantified as the percentage of RNA fragments smaller than 300 base pairs (Agilent Bioanalyzer, RNA 6000 Nano Kit). The qualification assessments (RNA quantity or quality) were informative but not used as acceptance criteria, except for samples that did not meet the minimum RNA input requirements. When needed, overdiluted RNA was concentrated using the clean-up approach from QIAGEN RNeasy kit protocols. Good sample quality was defined as <50% of RNA fragments of 50–300 base pairs in size. All the extracted RNA was tested independent of the concentration or the degradation rate. One RNA QC sample was included in each testing run as a positive control for extraction.

RNA expression profiling was performed using one standard panel (PanCancer human Immuno-Oncology 360 Panel) and two custom NanoString panels (Supplementary Tables 4 and 5). After data normalization and analysis, a high or low Immunosign score cutoff was arbitrarily defined as the 25th percentile of the observed scores among samples, and gene expression levels for 21 pre-specified genes were compositely scored as Immunosign 21 (Extended Data Fig. 6a). High scores indicated expression of immune-related genes that were potentially associated with tumor response. Univariate and multivariate analyses were performed to determine whether pre-treatment tumor or immune features influence clinical responses.

The determination of the COO subtypes of DLBCL was performed following transcriptomic^71^ (lymph2Cx) or proteomic (Hans algorithm) methods in place in patient care centers.

### IHC, Immunoscore TL, Immunoscore TCE, Immunoscore TCE^+^ and Immunoscore SC

H&E staining allowed preliminary tissue evaluation for FFPE block QC. Slides were scanned with the NanoZoomer-XR to generate digital images (×20). A pathologist identified the tumor area and provided qualitative and semi-quantitative assessments. CD19 IHC staining (LE-CD19) was scored by composite H-score (0–5 = ‘No’; 6–300 = ‘Yes’).

Results of the Immunoscore TL assay, which measures the density of CD8^+^ Tc cells and CD3^+^ T cells in resected or biopsied cancer samples, are expressed as a score determined by a percentile approach^72^. Consecutive FFPE slices (4 µm) were immunostained using a qualified BenchMark XT in accordance with the following workflow and reagents: antigen retrieval; staining with primary antibody CD3, HDX2 or CD8, HDX1 or HalioDx/Veracyte SAS; detection with a secondary antibody using an ultraView Universal DAB Detection Kit (Roche, 760-500); and counterstaining using the hematoxylin and bluing reagent Hematoxylin II (Roche, 790-2208). Control slides were systematically included in each staining run to permit QC of the obtained measurements. After coverslipping, slides were scanned with the NanoZoomer-XR to generate digital images (×20) and were analyzed in parallel by two independent, qualified operators. CD3 and CD8 IHC staining (Extended Data Fig. 3ab) was scored and converted into an Immunoscore using the HalioDx/Veracyte algorithm.

The Immunoscore TCE (CD3, CD8, FoxP3, PD-1, LAG-3 and TIM-3; Extended Data Fig. 3c and Supplementary Table 9) and SC (CD11b, CD14, CD15, CD68, S100A9 and LOX-1; Extended Data Fig. 3d and Supplementary Table 9) sequential IHC panels were performed to measure 14 myeloid and T cell subsets using FFPE biopsy specimens. The Immunoscore TCE^+^ sequential IHC panel (CD3, CD8, FoxP3, PD-1, LAG-3, TIM-3, TOX and EZH2; Extended Data Figs. 3 and 4 and Supplementary Table 9) was performed to measure 37 T cell subsets (Supplementary Table 9). Phenotype consistency between TCE and TCE^+^ stainings was demonstrated using eight samples that overlapped between the TCE and TCE^+^ panels (Extended Data Fig. 5c). Successive stainings were performed on the same slide using a Leica Bond RX and the antibodies listed in Supplementary Table 13. For the TCE and TCE^+^ panels, signal detection was performed using MACH 2 rabbit universal horseradish peroxidase polymer or MACH 2 mouse universal horseradish peroxidase polymer as secondary antibody and ImmPACT AMEC Red substrate detection. Counterstaining of cellular nuclei using hematoxylin was performed at the end of each staining workflow. One control slide was systematically included in each run to permit QC of the obtained measurements using qualitative acceptance criteria (specificity, staining location (nucleus/membrane), cell type and lack of background or unspecific staining). After each individual staining, coverslipping was performed automatically by the workstation CTM6 with aqueous mounting. Slides were scanned with the NanoZoomer-XR (×20), and a visual QC permitted qualification. Coverslips were carefully removed from slides using a warm water bath; slides were AMEC-destained by ethanol; and antibodies were stripped with denaturing solution. Each sample was analyzed using a HalioDx Digital Pathology Platform. Images were aligned with Brightplex-fuse (in-house software). Tumor areas were identified using annotation tools; subsequently, positively stained cells were detected and quantified in the selected regions of interest using HALO software (Indica Labs). Phenotypes of stained cells were visually verified according to expected staining and analyzed with Brightplex MultiplexR (in-house software).

### Statistics and reproducibility

No valid data were excluded from the analyses.

All statistical analysis and graphics were performed using R (version 4.0.3) and R Studio (version 1.3.1093) software. *t*-tests, exact Fisher tests, Wilcoxon tests, Kruskal–Wallis tests and Spearman correlation tests were evaluated using R built-in stats library. Heat maps were performed using the library ComplexHeatmap (version 2.4.3); box plots and scatter plots were performed using the library ggpubr (version 0.4.0); Cox proportional hazards regression was performed using the library survival (version 3.2.7); and Kaplan–Meier plots were performed using the library survminer (version 0.4.8). For each box plot, the lower bond, center and higher bond of the box are 25th (Q1), 50th (median) and 75th (Q3) percentiles, respectively. The boundaries of the whiskers are found within the 1.5 interquartile range (IQR) value (where IQR = Q3 − Q1). From above the upper quartile (Q3), a distance of 1.5 times the IQR is measured out, and a whisker (max.whisker) is drawn up to the largest observed data point from the dataset that falls within this distance. Similarly, a distance of 1.5 times the IQR is measured out below the lower quartile (Q1), and a whisker (min.whisker) is drawn down to the lowest observed data point from the dataset that falls within this distance. The box plot statistics are shown in Supplementary Table 14.

### Reporting summary

Further information on research design is available in the Nature Research Reporting Summary linked to this article.

## Online content

Any methods, additional references, Nature Research reporting summaries, source data, extended data, supplementary information, acknowledgements, peer review information; details of author contributions and competing interests; and statements of data and code availability are available at 10.1038/s41591-022-01916-x.

## Supplementary information

Supplementary Information Supplementary Legend Figs. 1–4, Supplementary Figs. 1-4, Supplementary Legend Tables 1–14 and Supplementary Tables 1, 2, 3, 4, 5, 11 and 13

Reporting Summary

Excel Workbook 1 Large Supplementary Tables

## Data Availability

Clinical response data and demographics/patient characteristics for ZUMA-1 are available in Supplementary Table 1, and demographics/patient characteristics for ZUMA-7 are available in Supplementary Table 2. Patient-related data not included in the paper were generated as part of clinical trials and may be subject to patient confidentiality. Any data and materials that can be shared will be released via a material transfer agreement. Correlations between pre-treatment gene expression of cytokines and cytokine-responsive transcription factors and T cell markers in ZUMA-1 patients who achieved CR are available in Supplementary Table 6. Correlations between pre-treatment gene expression of cytokines and cytokine-responsive transcription factors and T cell markers in ZUMA-1 patients who achieved OR are available in Supplementary Table 7. Correlations between pre-treatment gene expression of cytokines and cytokine-responsive transcription factors and T cell markers in ZUMA-1 patients who did not achieve CR or OR are available in Supplementary Table 8. Differential gene expression by pathway in pre-treatment tumor biopsies of axi-cel responders versus non-responders are available in Supplementary Table 12. The NanoString data from ZUMA-1 patients discussed in this publication have been deposited in the National Center of Biotechnology Information Gene Expression Omnibus (GEO) and are accessible through GEO Series with the following accession number and access code: GSE197977 and chuhmyuidlwrdiv.
