# Supplementary material for: Tumor immune contexture is a determinant of anti-CD19 CAR T cell efficacy in large B cell lymphoma
Source: Nat Med. 2022 Aug 29;28(9):1872–82. doi: 10.1038/s41591-022-01916-x (PMC9499856; doi:10.1038/s41591-022-01916-x)
Supplement: Supplementary file 1 — Supplementary Legend Figs. 1–4, Supplementary Figs. 1-4, Supplementary Legend Tables 1–14 and Supplementary Tables 1, 2, 3, 4, 5, 11 and 13 [file 41591_2022_1916_MOESM1_ESM.pdf]

nature portfolio

https://doi.org/10.1038/s41591-022-01916-x

Supplementary information

Tumor immune contexture is a determinant of anti-CD19 CAR T cell efficacy in large B cell lymphoma

In the format provided by the authors and unedited

Tumour immune contexture is a determinant of anti-CD19 CAR T-cell efficacy in large B-cell lymphoma

Authors: Nathalie Scholler[1,2], Regis Perbost[3], Frederick L. Locke[4], Michael D. Jain[4], Sarah Turcan[3], Corinne Danan[3], Edmund C. Chang[1], Sattva S. Neelapu[5], David B. Miklos[6], Caron A. Jacobson[7], Lazaros J. Lekakis[8], Yi Lin[9], Armin Ghobadi[10], Jenny J. Kim[1], Justin Chou[1], Vicki Plaks[1], Zixing Wang[1], Allen Xue[1], Mike Mattie[1], John M. Rossi[1], Adrian Bot[1,11] & Jérôme Galon[3,12]*

Affiliations: [1]Kite, a Gilead Company, Santa Monica, CA, USA; [2]Gilead Sciences, Foster City, CA, USA; [3]Veracyte SAS, Marseille, France; [4]Moffitt Cancer Center, Tampa, FL, USA; [5]MD Anderson Cancer Center, Houston, TX, USA; [6]Stanford University School of Medicine, Stanford, CA, USA; [7]Dana-Farber Cancer Institute, Boston, MA, USA; [8]University of Miami Health System, Sylvester Comprehensive Cancer Center, Miami, FL, USA; [9]Mayo Clinic, Rochester, MN, USA; [10]Washington University School of Medicine, St. Louis, MO, USA; [11]Capstan Therapeutics, San Diego, CA 92121;[12]INSERM, Sorbonne Université, Université de Paris, Centre de Recherche des Cordeliers, Equipe Labellisée Ligue Contre le Cancer, Laboratory of Integrative Cancer Immunology, F-75006 Paris, France;

*Corresponding author:

Jérôme Galon, PhD

Laboratory of Integrative Cancer Immunology

INSERM, Cordeliers Research Center

15 rue de l'École de Médecine, 75006, Paris, France

Phone: +33 1 44 27 9085

E-mail: jerome.galon@crc.jussieu.fr

Supplementary Figures

Supplementary Fig. 1. Correlative studies of clinical outcomes with pretreatment TME scores, cell subsets and gene expression. **a,b** Correlation of Immunoscore TL (Digital pathology) with Immunosign 21 prespecified gene panel in pretreatment TME of **a,** ZUMA-1 patients (n=24) by response (CR [n=17] vs. PR/SD/PD [n=7]) or neurologic event grade (grade 0-2 vs. ≥ 3); and **b,** treatment-naïve DLBCL patients (n=50); the grey ribbons show the 95% Confidence Interval of the regression line and the statistical significance of the spearman coefficient level (two-sided P value) is shown in the panels. **c-j** Correlation of ZUMA-1 patient clinical outcomes with prespecified indexes derived from the analysis of pretreatment tumour biopsies with digital pathology (Immunoscore) and transcriptomics (IS21). Correlations of Immunoscore (**c-f)** and IS21 (**g-j**) with CR (**c**, **g**), CR/PR (**d, h**), PR/SD/PD (**e**, **i**), or SD/PD (**f, j**). Immunoscore CR (high n = 14, low n = 7) vs. nonCR (high n = 4, low n = 5), *P* = 0.418; Immunoscore CR/PR (high n = 16, low n = 9) vs. SD/PD (high n = 2, low n = 3), *P* = 0.364; IS21 CR (high n = 16, low n = 4) vs. nonCR (high n = 5, low n = 4), *P* = 0.209; IS21 CR/PR (high n = 18, low n = 4) vs. SD/PD (high n = 3, low n = 4), *P* = 0.0.68; *P* values were derived with a two-sided exact Fisher test.

Supplementary Fig. 2. Correlative studies of pretreatment myeloid subsets in ZUMA-1 tumour biopsies with post axi-cel neurologic events. Densities of T-cell subsets (cells/mm^2) were quantified in ZUMA-1 pretreatment tumour biopsies (subset 1) with Immunoscore SC panels and plotted as functions of clinical responses or NE grade. **a,b.** Myeloid cell subset densities in association with clinical outcomes; PR/SD/PD (n=7) versus CR (n=10) or SD/PD (n=4) versus CR/PR (n=13). P values were derived with a two-sided Wilcoxon test. **c,d.** Myeloid cell phenotype in association with NE grades; grades 0–2 (n=14) versus grade ≥3 (n=4), (**c**) P values were derived with a two sided Wilcoxon test; (**d**) P values were derived with a two sided t.test without adjustment. CR, complete response; M-MDSC, monocytic myeloid-derived suppressor cell; NE, neurologic event; PD, progressive disease; PMN-MDSC, polymorphonuclear myeloid-derived suppressor cell; PR, partial response; SC, Suppressive Cell; SD, stable disease.

Supplementary Fig. 3. Correlative studies of pretreatment T-cell subsets in ZUMA-1 tumour biopsies with post axi-cel neurologic events. Densities of T-cell (cells/mm^2) were quantified in ZUMA-1 pretreatment tumour biopsies (subset 1) and plotted as functions of clinical responses or NE grade. **a,** Cell densities of Treg (cells/mm^2) (n=27) were plotted as functions of NE grade after axi-cel infusion (grade 0–2 versus grade ≥3) and of the number of immune checkpoints expressed (0–3; PD-1, LAG-3, and/or TIM-3). *P* values were derived from two-sided Wilcoxon test. **b,** Correlations (Spearman R) of cell densities of Treg with other T-cell subsets (cells/mm^2) (n=27); grades 0–2 (n=14) versus grade ≥3 (n=4). The grey ribbons show the 95% Confidence Interval of the regression line.. NE, neurologic event; IC, immune checkpoint; Tc, cytotoxic T-cell; Th, helper T-cell; Treg, regulatory T-cell

Supplementary Fig. 4. Proposed model linking the pretreatment TME with clinical response to axi-cel.

Pretreatment tumour biology features supporting a TME rich in select chemokines (CCL5, CXCL9), γ-chain receptor cytokines (IL-7, IL-15), and IFN-regulated molecules help favour recruitment and activation of tumour-infiltrating T-cells, thereby facilitating clinical response to axi-cel following infusion. The TME gene expression profile of axi-cel responders evolves rapidly toward an activated T-cell–related signature paralleled by a decreasing tumour-related signature, markedly differing from the pattern observed in nonresponders. At relapse, the TME evolves once more, but toward an immune-detrimental contexture, with a decreased T-cell signature and increased counterregulatory molecules. Furthermore, the pretreatment TME differed between patients who developed and those who did not develop high-grade NEs. Patients with grade ≥3 neurotoxicity had reduced infiltration of Tregs within the pretreatment TME, suggesting a protective role for Tregs against toxicity without an apparent impact on response in relapsed/refractory large B-cell lymphoma**.**

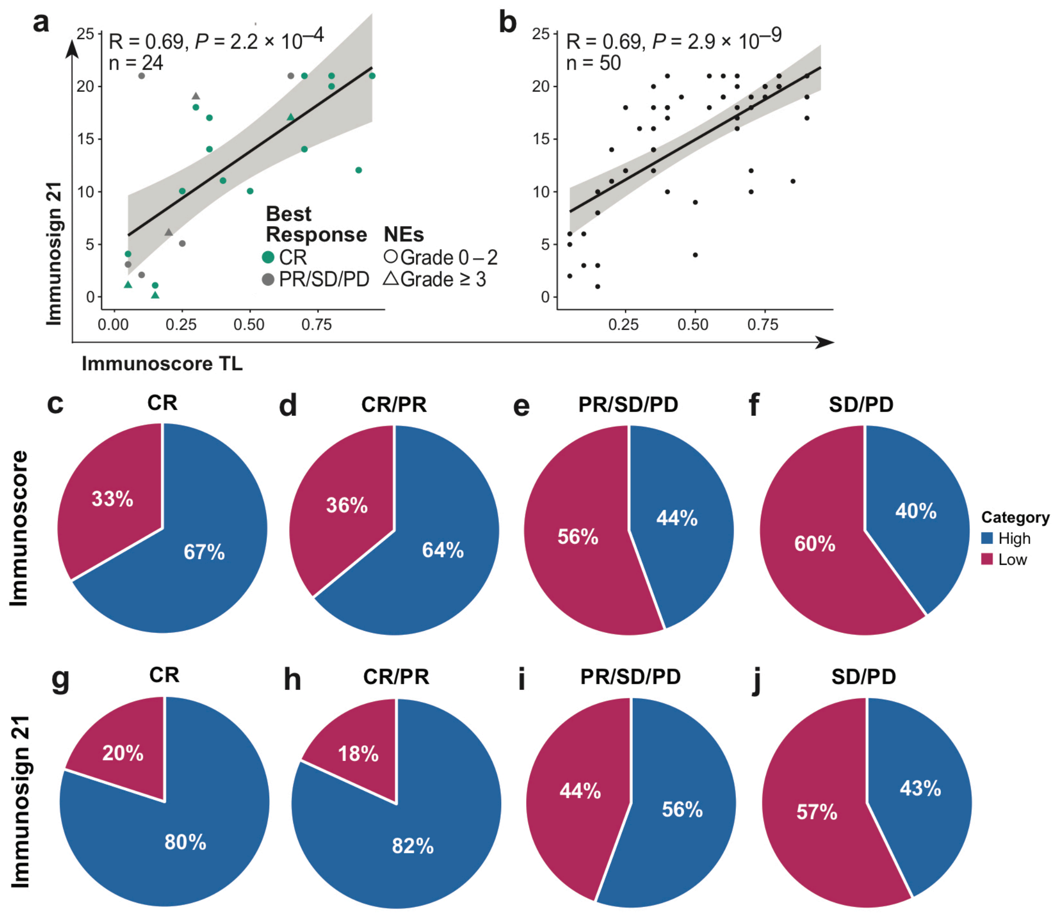
a
R = 0.69, P = 2.2 × 10−4
n = 24
Immunosign 21
Best Response
CR
PR/SD/PD
NEs
Grade 0 – 2
Grade ≥ 3
b
R = 0.69, P = 2.9 × 10−9
n = 50
Immunoscore TL
c
CR
33%
67%
d
CR/PR
36%
64%
e
PR/SD/PD
56%
44%
f
SD/PD
60%
40%
Immunoscore
Category
High
Low
g
CR
20%
80%
h
CR/PR
18%
82%
i
PR/SD/PD
44%
56%
j
SD/PD
57%
43%
Immunosign 21

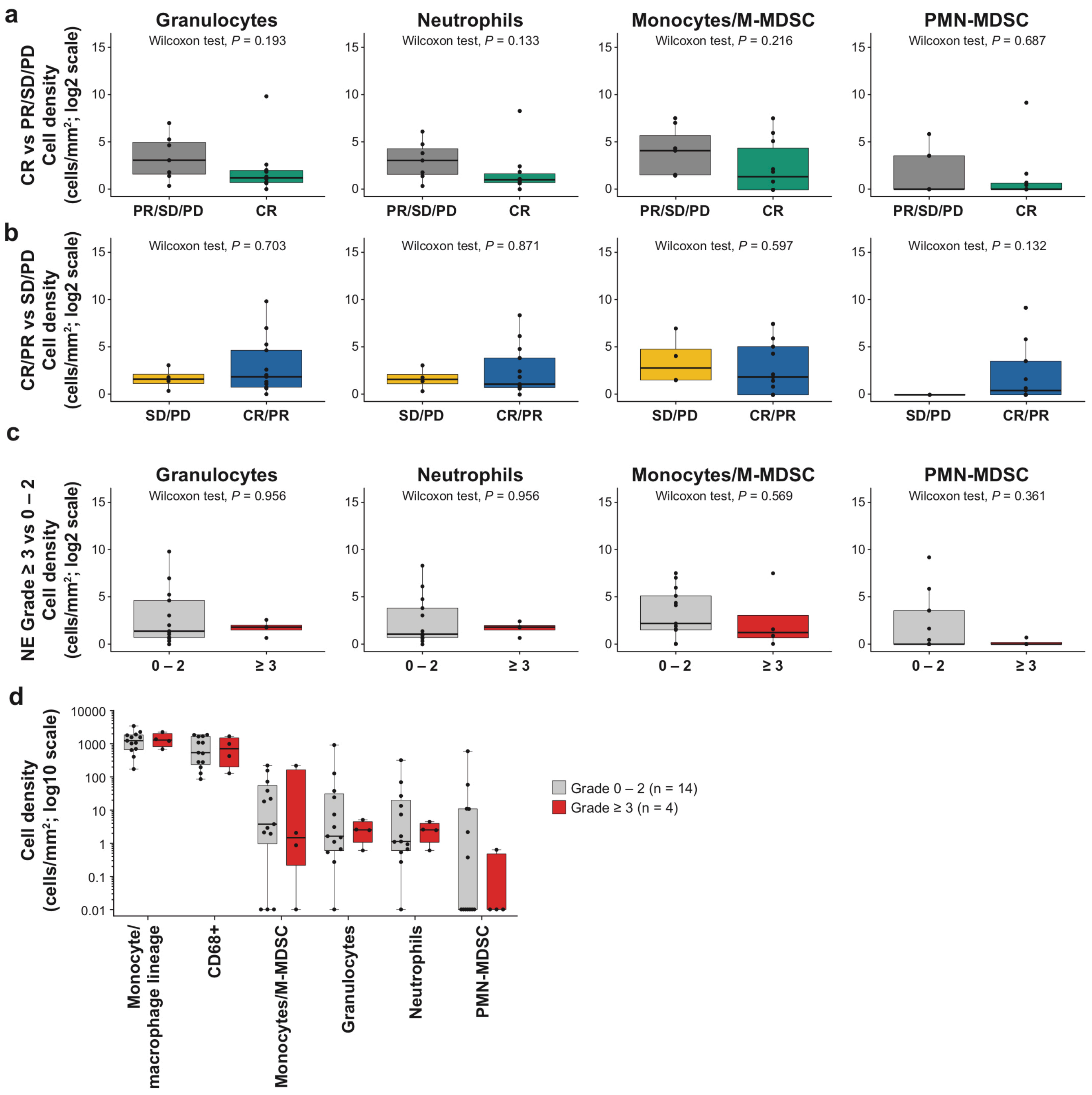

a
CR vs PR/SD/PD
Cell density
(cells/mm2; log2 scale)
Granulocytes
Wilcoxon test, P = 0.193
Neutrophils
Wilcoxon test, P = 0.133
Monocytes/M-MDSC
Wilcoxon test, P = 0.216
PMN-MDSC
Wilcoxon test, P = 0.687
PR/SD/PD
CR
b
CR/PR vs SD/PD
Cell density
(cells/mm2; log2 scale)
Wilcoxon test, P = 0.703
Wilcoxon test, P = 0.871
Wilcoxon test, P = 0.597
Wilcoxon test, P = 0.132
SD/PD
CR/PR
c
NE Grade ≥ 3 vs 0 – 2
Cell density
(cells/mm2; log2 scale)
Granulocytes
Wilcoxon test, P = 0.956
Neutrophils
Wilcoxon test, P = 0.956
Monocytes/M-MDSC
Wilcoxon test, P = 0.569
PMN-MDSC
Wilcoxon test, P = 0.361
0 – 2
≥ 3
d
Cell density
(cells/mm2; log10 scale)
10000
1000
100
10
1
0.1
0.01
Monocyte/
macrophage lineage
CD68+
Monocytes/M-MDSC
Granulocytes
Neutrophils
PMN-MDSC
Grade 0 – 2 (n = 14)
Grade ≥ 3 (n = 4)

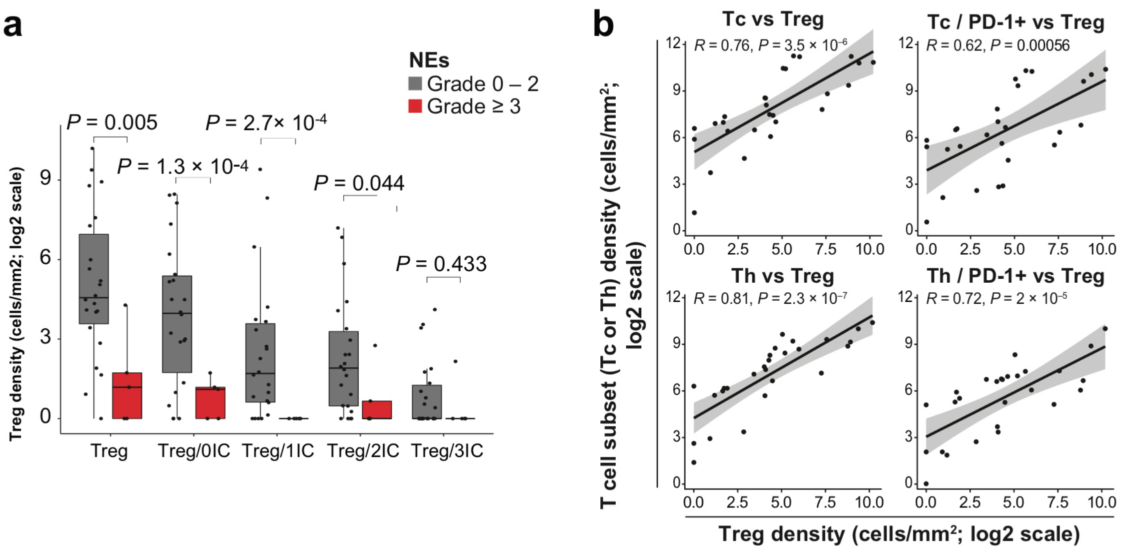

a
NEs
Grade 0 – 2
Grade ≥ 3
P = 0.005
P = 1.3 × 10-4
P = 2.7× 10-4
P = 0.044
P = 0.433
Treg density (cells/mm2; log2 scale)
Treg
Treg/0IC
Treg/1IC
Treg/2IC
Treg/3IC
b
Tc vs Treg
R = 0.76, P = 3.5 × 10-6
Tc / PD-1+ vs Treg
R = 0.62, P = 0.00056
Th vs Treg
R = 0.81, P = 2.3 × 10-7
Th / PD-1+ vs Treg
R = 0.72, P = 2 × 10-5
T cell subset (Tc or Th) density (cells/mm2; log2 scale)
Treg density (cells/mm2; log2 scale)

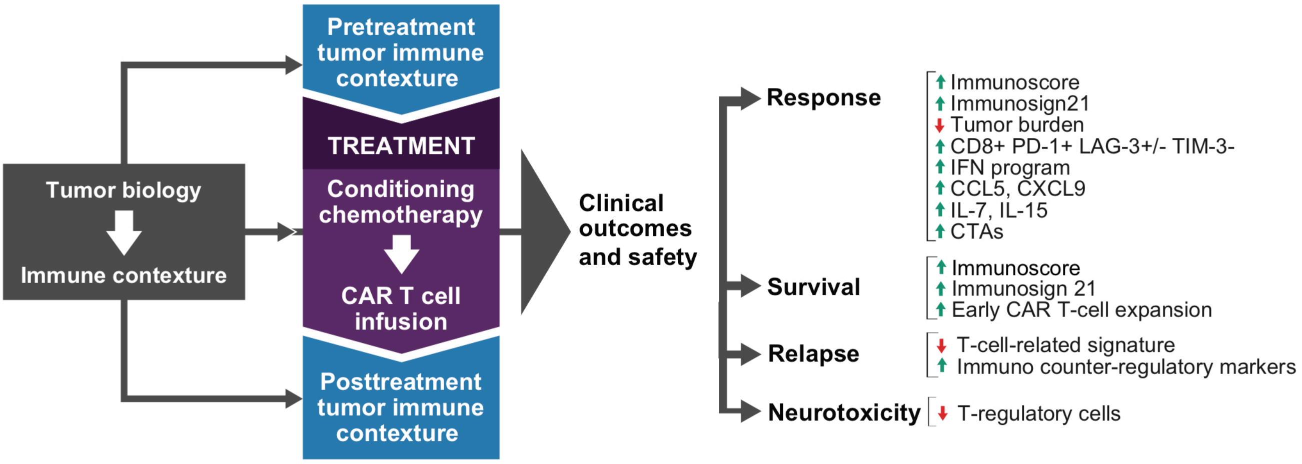

Pretreatment tumor immune contexture
TREATMENT
Conditioning chemotherapy
CAR T cell infusion
Posttreatment tumor immune contexture
Tumor biology
Immune contexture
Clinical outcomes and safety
Response
↑ Immunoscore
↑ Immunosign21
↓ Tumor burden
↑ CD8+ PD-1+ LAG-3+/- TIM-3-
↑ IFN program
↑ CCL5, CXCL9
↑ IL-7, IL-15
↑ CTAs
Survival
↑ Immunoscore
↑ Immunosign 21
↑ Early CAR T-cell expansion
Relapse
↓ T-cell-related signature
↑ Immuno counter-regulatory markers
Neurotoxicity
↓ T-regulatory cells

Supplementary Table 2. Demographics and baseline characteristics of Zuma 7 subjects with Immunosign and T cell-associated gene expression

Data cutoff date = 18MAR2021- Data Source: ADSL, ADBASE   Program Name: t_bchar.sas Abbreviations: BCL, B-cell lymphoma; DLBCL, diffuse large B-cell lymphoma; ECOG, Eastern Cooperative Oncology Group; HGBL, high-grade B-cell lymphoma; IXRS, interactive voice/web response system; LDH, lactate dehydrogenase; Max, maximum; Min, minimum; MYC, myelocytomatosis oncogene; NOS, not otherwise specified; Q1, first quartile; Q3, third quartile; SPD, sum of product of perpendicular diameters of up to 6 measurable target lesions; STDEV, standard deviation; ULN, upper limit of normal.

Note: HGBL – double hit is defined as presence of C-MYC and either BCL-2 or BCL-6 rearrangements; HGBL - triple hit is defined as presence of BCL-2, BCL-6, and C-MYC rearrangements.

Note: Baseline SPD is defined as the last value taken prior to randomization. If measurement prior to randomization is not available, the last value taken on or prior to axicabtagene ciloleucel infusion date/standard of care salvage chemotherapy Cycle 1 Day 1 is used.

Supplementary Table 6. Differential expression of all genes assessed pre- (baseline) and post–axi-cel infusion in ZUMA-1 patients who achieved CR.

Baseline refers to samples collected before conditioning chemotherapy and axi-cel infusion (subset 1). Posttreatment samples were collected early after CAR T-cell infusion (between days 7 and 14 after axi-cel treatment, subset 2). Due to the size of this table, please see the supplemental excel file entitled, "**Supplementary Table 6**," for these data. axi-cel, axicabtagene ciloleucel; CAR, chimeric antigen receptor; CR, complete response.

Supplementary Table 7. Differential expression of all genes assessed pre- (baseline) and post–axi-cel infusion in ZUMA-1 patients who achieved OR

Baseline refers to samples collected before conditioning chemotherapy and axi-cel infusion (subset 1). Posttreatment samples were collected early after CAR T-cell infusion (between days 7 and 14 after axi-cel, subset 2). Due to the size of this table, please see the supplemental excel file entitled, “**Supplementary Table 7**,” for these data. axi-cel, axicabtagene ciloleucel; CAR, chimeric antigen receptor; PR, partial response.

Supplementary Table 8. Differential expression of all genes assessed pre- (baseline) and post–axi-cel infusion in ZUMA-1 patients who did not achieved CR or OR.

Baseline refers to samples collected before conditioning chemotherapy and axi-cel infusion (subset 1). Posttreatment samples were collected early after CAR T-cell infusion (between days 7 and 14 after axi-cel treatment, subset 2). Due to the size of this table, please see the supplemental excel file entitled, "**Supplementary Table 8**," for these data. axi-cel, axicabtagene ciloleucel; CAR, chimeric antigen receptor; PD, progressive disease; SD, stable disease.

Supplementary Table 9. Composition of Immunoscore panels (TCE, TCE+, and SC) and resulting cell type marker signatres

Due to the size of this table, please see the supplemental excel file entitled, "**Supplementary Table 9**," for these data. CR, complete response; FoxP3, forkhead box P3; LAG-3, lymphocyte-activation gene 3; LOX-1, lectin-type oxidised LDL receptor 1; M-MDSC, monocytic myeloid-derived suppressor cell; PD, progressive disease; PD-1, programmed cell death protein 1; PMN-MDSC, polymorphonuclear myeloid-derived suppressor cell; PR, partial response; SC, Suppressive Cell; SD, stable disease; TCE, T cell Exhaustion; TIM-3, T cell immunoglobulin and mucin domain 3; TOX, thymocyte selection-associated high mobility group box; Treg, regulatory T-cell.

Supplementary Table 10. Correlations between pretreatment gene expression of cytokines and cytokine-responsive transcription factors, and T-cell markers

Due to the size of this table, please see the supplemental excel file entitled, "**Supplementary Table 10**," for these data. Gene expression was measured using the PanCancer Immune + ImmunosignImmunosign NanoString panel. BH refers to an adjustment of the *P* values by the Benjamini-Hochberg method.

Supplementary Table 12. Differential gene expression by pathway in pretreatment tumour biopsies of axi-cel responders versus nonresponders.

P values were derived from Wilcoxon test. Due to the size of this table, please see the supplemental excel file entitled, "**Supplementary Table 12**," for these data. axi-cel, axicabtagene ciloleucel; CTA, cancer testis antigen; IFN, interferon; TAA, tumour-associated antigen.

Supplementary Table 14. Boxplot statistics

Boxplot statistics are provided for Fig. 1b,c; Fig. 3a,b,c,d; Fig. 6a,b; and ED Fig. 2b,c,d,e,f. Center.box, center of the box (50th percentile). Due to the size of this table, please see the supplemental excel file entitled, "**Supplementary Table 14**," for these data. CR, complete response; Max box, higher bond of the box (75th percentile, Q3); Max. value, maximum value; Max. whisker, largest observed data point within the 1.5 IQR; Min.box, lower bond of the box (25th percentile, Q1); Min.value, minimum value; Min. whisker, lowest observed data point within the 1.5 Inter Quartile Range (IQR, Q3-Q1); n, number of samples; NE Gr, neurotoxicity grade; no CR, no complete response; No OR, no objective response; OR status, objective response; Tr. Status, Treatment Status.
