# Supplementary material for: Tumor immune contexture is a determinant of anti-CD19 CAR T cell efficacy in large B cell lymphoma
Source: Nat Med. 2022 Aug 29;28(9):1872–82. doi: 10.1038/s41591-022-01916-x (PMC9499856; doi:10.1038/s41591-022-01916-x)
Supplement: Supplementary file 2 — Reporting Summary [file 41591_2022_1916_MOESM2_ESM.pdf]

nature research

Corresponding author(s): Jérôme Galon; NMED-A111611D

Last updated by author(s): Jun 29, 2022

Reporting Summary

Nature Research wishes to improve the reproducibility of the work that we publish. This form provides structure for consistency and transparency in reporting. For further information on Nature Research policies, see our Editorial Policies and the Editorial Policy Checklist.

Statistics

For all statistical analyses, confirm that the following items are present in the figure legend, table legend, main text, or Methods section.

n/a	Confirmed	
☐	☒	The exact sample size (n) for each experimental group/condition, given as a discrete number and unit of measurement
☐	☒	A statement on whether measurements were taken from distinct samples or whether the same sample was measured repeatedly
☐	☒	The statistical test(s) used AND whether they are one- or two-sided<br>*Only common tests should be described solely by name; describe more complex techniques in the Methods section.*
☐	☒	A description of all covariates tested
☒	☐	A description of any assumptions or corrections, such as tests of normality and adjustment for multiple comparisons
☐	☒	A full description of the statistical parameters including central tendency (e.g. means) or other basic estimates (e.g. regression coefficient) AND variation (e.g. standard deviation) or associated estimates of uncertainty (e.g. confidence intervals)
☒	☐	For null hypothesis testing, the test statistic (e.g. F, t, r) with confidence intervals, effect sizes, degrees of freedom and P value noted<br>*Give P values as exact values whenever suitable.*
☒	☐	For Bayesian analysis, information on the choice of priors and Markov chain Monte Carlo settings
☒	☐	For hierarchical and complex designs, identification of the appropriate level for tests and full reporting of outcomes
☐	☒	Estimates of effect sizes (e.g. Cohen's d, Pearson's r), indicating how they were calculated

Our web collection on statistics for biologists contains articles on many of the points above.

Software and code

Policy information about availability of computer code

Data collection	Not applicable
Data analysis	Not applicable

For manuscripts utilizing custom algorithms or software that are central to the research but not yet described in published literature, software must be made available to editors and reviewers. We strongly encourage code deposition in a community repository (e.g. GitHub). See the Nature Research guidelines for submitting code & software for further information.

Data

Policy information about availability of data

All manuscripts must include a data availability statement. This statement should provide the following information, where applicable:

- Accession codes, unique identifiers, or web links for publicly available datasets
- A list of figures that have associated raw data
- A description of any restrictions on data availability

The authors declare that the data supporting the findings of this study are available within the paper and its supplementary information files. Requests for raw data can be made through the corresponding author.

Field-specific reporting

Please select the one below that is the best fit for your research. If you are not sure, read the appropriate sections before making your selection.

☒ Life sciences ☐ Behavioural & social sciences ☐ Ecological, evolutionary & environmental sciences

For a reference copy of the document with all sections, see nature.com/documents/nr-reporting-summary-flat.pdf

Life sciences study design

All studies must disclose on these points even when the disclosure is negative.

Sample size	Tumour biopsies were collected from patients treated with axicabtagene ciloleucel (axi-cel) in the ZUMA-1 pivotal study. Tumour biopsies were performed at baseline (before conditioning chemotherapy and axi-cel infusion), early after CAR T cell infusion (Day 7 – Day 14), or later at relapse. All samples that passed quality control were included in this study, without exception.
Data exclusions	No data were excluded from the analyses.
Replication	Multiple samples were included in each analysis. However, as noted in the Discussion, the findings reported in the study could be confirmed by prospective validation in subsequent trials.
Randomization	Samples were not allocated to specific groups but were rather analyzed based on when the tumour biopsy was collected (ie, baseline, early after CAR T cell infusion, or at relapse).
Blinding	Each sample analysed in the study had a unique identifier that was used during data collection. The investigators did have access to the code detailing which samples belong to which patient, so a formal blinding process was not in place.

Reporting for specific materials, systems and methods

We require information from authors about some types of materials, experimental systems and methods used in many studies. Here, indicate whether each material, system or method listed is relevant to your study. If you are not sure if a list item applies to your research, read the appropriate section before selecting a response.

Materials & experimental systems

n/a	Involved in the study	
☐	☒	Antibodies
☒	☐	Eukaryotic cell lines
☒	☐	Palaeontology and archaeology
☒	☐	Animals and other organisms
☐	☒	Human research participants
☐	☒	Clinical data
☒	☐	Dual use research of concern

Methods

n/a	Involved in the study	
☒	☐	ChIP-seq
☒	☐	Flow cytometry
☒	☐	MRI-based neuroimaging

Antibodies

Antibodies used	The antibodies, reagents, and workflow used for the immunohistochemistry analyses in this study are detailed within the "Immunohistochemistry, Immunoscore TL, Immunoscore TCE, and Immunoscore SC" section of the Methods.
Validation	Not applicable

Human research participants

Policy information about studies involving human research participants

Population characteristics	Patient characteristics and clinical outcomes described in this study were consistent with those observed in the overall ZUMA-1 cohort. LBCL and ZUMA-7 patients at baseline were also used for mechanistic studies.
Recruitment	Patients were enrolled in ZUMA-1 and ZUMA-7 following screening and confirmation of eligibility. Tumour biopsies were collected from patients treated with axi-cel in ZUMA-1 and ZUMA-7. All samples that passed quality control were included in this study, without exception.
Ethics oversight	The study was approved by the institutional review board at each study site and was conducted in accordance with the Good Clinical Practice guidelines of the International Conference on Harmonisation.

Note that full information on the approval of the study protocol must also be provided in the manuscript.

April 2020

Clinical data

Policy information about clinical studies

All manuscripts should comply with the ICMJE guidelines for publication of clinical research and a completed CONSORT checklist must be included with all submissions.

Clinical trial registration	NCT02348216 (ZUMA-1)
Study protocol	The study protocol is available as part of the original disclosure of the Phase 1/2 data in the Neelapu et al. New Engl J Med. 2017;377(26):2531-2544.
Data collection	Tumour biopsies were collected from patients treated with axi-cel in the ZUMA-1 and ZUMA-7 pivotal study and were analyzed in this study.
Outcomes	Outcomes were not assessed in this study prospectively. However, patient responses during time on study in ZUMA-1 were collected retrospectively.
